# Optimal intervention strategies to mitigate the COVID-19 pandemic effects

**DOI:** 10.1038/s41598-022-09857-8

**Published:** 2022-04-12

**Authors:** Andreas Kasis, Stelios Timotheou, Nima Monshizadeh, Marios Polycarpou

**Affiliations:** 1 Department of Electrical and Computer Engineering, KIOS Research and Innovation Center of Excellence, University of Cyprus, Nicosia, Cyprus; 2 Engineering and Technology Institute, University of Groningen, Nijenborgh 4, 9747AG Groningen, The Netherlands

**Keywords:** Computational models, Epidemiology, Applied mathematics

## Abstract

Governments across the world are currently facing the task of selecting suitable intervention strategies to cope with the effects of the COVID-19 pandemic. This is a highly challenging task, since harsh measures may result in economic collapse while a relaxed strategy might lead to a high death toll. Motivated by this, we consider the problem of forming intervention strategies to mitigate the impact of the COVID-19 pandemic that optimize the trade-off between the number of deceases and the socio-economic costs. We demonstrate that the healthcare capacity and the testing rate highly affect the optimal intervention strategies. Moreover, we propose an approach that enables practical strategies, with a small number of policies and policy changes, that are close to optimal. In particular, we provide tools to decide which policies should be implemented and when should a government change to a different policy. Finally, we consider how the presented results are affected by uncertainty in the initial reproduction number and infection fatality rate and demonstrate that parametric uncertainty has a more substantial effect when stricter strategies are adopted.

## Introduction

A novel coronavirus was first reported in Wuhan, China in December 2019^1^. The virus, now known as severe acute respiratory coronavirus 2 (SARS-CoV-2)^2^, spread rapidly through China and the rest of the world causing the coronavirus disease 2019 (COVID-19), being officially declared a pandemic by the World Health Organization (WHO) on March 17th, 2020. Since the outbreak of the COVID-19 pandemic, the world has been facing an unprecedented human tragedy along with fears of economic devastation. As a result, more than 450 million infected cases and 6 million deaths have been reported to this date (March 15, 2022). To cope with the effects of the virus, governments across the world have implemented a range of non-pharmaceutical interventions such as closing schools, banning public events and imposing social distancing, self-isolation and lockdown policies. Although such interventions may curtail the infection rate of the disease and hence the spread of the virus^3,4^, they impose an enormous economic effect. According to the International Monetary Fund^5^, the economic impact of the pandemic is expected to cause the steepest worldwide recession in over 40 years and result in a loss of more than 5% of the gross domestic product in the developed world. Hence, although a combination of social distancing and lockdown policies may be effective in containing the virus, it might be highly costly in terms of economical impact, which naturally makes government decision making a multi-objective problem.

Mathematical models are fundamental to describe the dynamic evolution of pandemics and to form effective policies to mitigate their impact. A seminal study in this area is Ref.^6^, which describes the widely used susceptible-infected-recovered (SIR) model. A comprehensive review of epidemiology models can be found in Ref.^7^. Such models enable the study of the progression of various diseases over time, and facilitate the characterization of their asymptotic behaviour and dependence on model parameters. Recently, there have been various approaches to model the progression of the COVID-19 outbreak. A common approach is to apply different extensions of the SIR model, e.g.^8^. A time-varying susceptible-infected-recovered-deceased model has been proposed in Ref.^9^. In addition, a more involved compartmental model has been developed in Ref.^10^, offering larger modelling flexibility compared to simpler models. Furthermore, Ref.^11^ developed an extended model which took into account the regional heterogeneity of the pandemic.

Two important parameters in the study of epidemic progression are the basic reproduction number and the infection fatality rate. The former is interpreted as the number of new people that the average person transmits the disease to while the latter enables an estimate of the fatalities resulting from the disease. The initial reproduction number, i.e. the basic reproduction number at the onset of the disease, which we denote by $\overline{R}_0$, is also of particular importance to accurately model the disease progression and in deciding the extend of government policies. However, there is significant uncertainty in estimating these parameters, as demonstrated via numerous studies that estimate $\overline{R}_0$ using statistical data from different countries^12–15^, and various studies that have reported different infection fatality rates^16–18^. Hence, it is important to consider the effect of parametric uncertainty in forming effective government strategies.

## Contribution

This study uses tools from optimal control theory to address the problem of forming a practical and efficient government intervention strategy that limits the number of fatalities due to the COVID-19 pandemic with a low social and economic cost until a vaccine is fully deployed.

In particular, we consider a controlled SIDARE (Susceptible, Infected undetected, infected Detected, Acutely symptomatic—threatened, Recovered, deceased—Extinct) model that takes into account the effect of government intervention policies. The considered model enables the integration of features such as the impact of the available healthcare capacity and testing rate. The contribution of this study is summarized as follows:

(i) *Fatalities versus economic cost.* We present the relation between the number of fatalities and cost of optimal government intervention, and study how this relation is affected by the amount of testing and the capacity of the healthcare system to treat patients. We demonstrate the effect of these parameters in the decease rate of the pandemic and the resulting cost associated with the optimal intervention strategy. In addition, for a range of adopted decease tolerance levels, we provide insights on the shape of the optimal intervention strategy and its dependence on the adopted test policy.
(ii) *Which policies and when.* We consider the fact that a government can only implement a limited number of policies and policy changes over the time span of the pandemic, due to practicality and implementability reasons and to avoid the social fatigue resulting from frequent changes in policy. Our approach provides tools to decide which policies should be implemented and when should a government change to a different policy. We demonstrate that a small number of policies and policy changes yields a close to optimal government strategy. In particular, our results suggest that the additional cost incurred from implementing 4 policies and 6 policy changes is less than 1% compared to the optimal continuously changing strategy.
(iii) *Impact of uncertainty.* We consider the impact of uncertainty in the value of the initial basic reproduction number $\bar{R}_0$ and the infection fatality rate on the decease rates resulting from optimal government strategies associated with particular decease tolerance levels. We demonstrate that parametric uncertainty has a larger impact when stricter government policies, associated with lower decease tolerances, are adopted.

## Results

### Problem description

**Figure 1:**
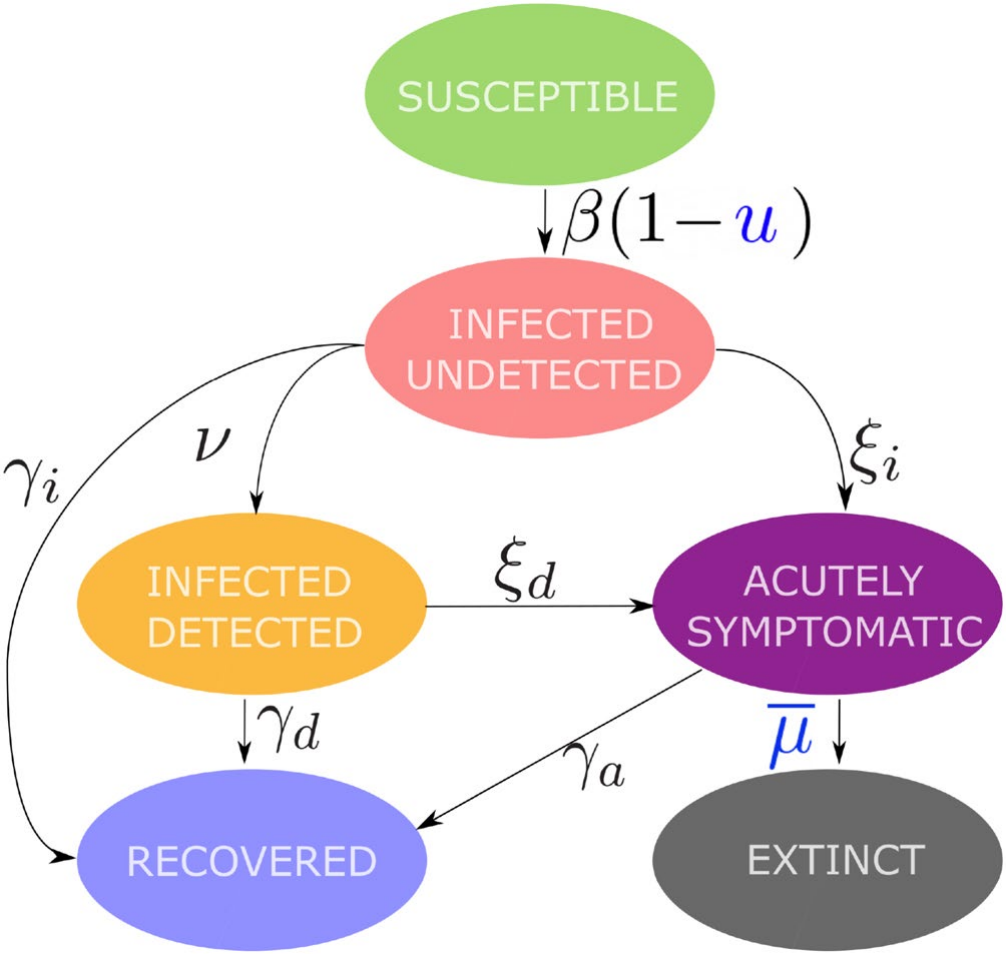
The controlled SIDARE model. Schematic representation of the controlled SIDARE model, used to describe the evolution of the COVID-19 pandemic. The model splits the population into Susceptible, Infected undetected, infected Detected, Acutely symptomatic—threatened, Recovered and deceased—Extinct. Model parameters $\beta , \xi _i, \xi _d, \nu , \gamma _i, \gamma _d$ and $\gamma _a$ describe the transition rates between the states. The effect of government interventions is described by *u* which limits the rate of infection. The rate at which the acutely symptomatic population deceases is described by $\overline{\mu }$, which depends on the healthcare system capacity.

To study the progression of the pandemic, we consider a controlled SIDARE (Susceptible, Infected undetected, infected Detected, Acutely symptomatic—threatened, Recovered, deceased—Extinct) model (see Fig. 1), where the effects of the healthcare capacity limit and non-pharmaceutical government interventions on the mortality and infection rates are taken into account. A full mathematical description of the controlled SIDARE model and explanations on its components are provided in the “Methods” section. Note that the terms threatened and acutely symptomatic, as well as deceased and extinct are used interchangeably. In addition, we form a multi-objective optimization problem whose cost function consists of three components: (i) the socio-economic cost of government intervention, (ii) the cost associated with hospitalization and medical care of the acutely symptomatic population and (iii) a cost proportional to the portion of the deceased population. We seek intervention strategies that minimize the aforementioned cost function. We then investigate how such strategies can be obtained with a limited number of distinct policies and policy changes in the described optimization problem. Further motivation and details concerning the mathematical formulation of the optimization problem are provided in the “Methods” section, while our approach to solve it is detailed in the Supplementary Information (SI) (see Optimal control methodology).

**Figure 2:**
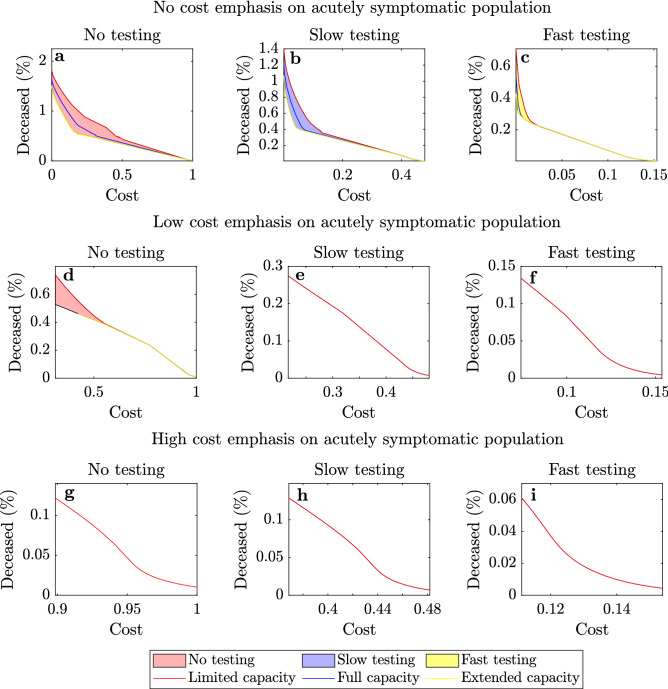
Deceased population vs. cost. Proportion of deceased population versus cost of optimal government intervention when the available healthcare capacity for COVID-19 patients is limited (red), full (blue), and extended (yellow) and when no testing (**a,d,g**), slow testing (**b,e,h**) and fast testing (**c,f,i**) policies are adopted. In addition, we present the cases where no emphasis (**a–c**), low emphasis (**d–f**) and high emphasis (**g–i**) is given to the cost associated with the acutely symptomatic population. When identical relations are obtained for different healthcare capacity levels, as in (**e–i**), then only the lowest capacity is presented. Shaded regions show the ranges of the relations between deceased population percentage and cost of government interaction when the healthcare capacity is between the limited and extended levels. All presented costs are normalised using as basis the cost of the optimal government strategy with no testing resulting to 0.01% deceases.

### Deceased population versus cost of government intervention

In this section we study the problem of forming an optimal government intervention strategy and its dependence on selected parameters. In particular, we consider the impact of (i) the healthcare capacity limit, (ii) the testing rate and (iii) the cost emphasis on the acutely symptomatic and deceased population, on the optimal intervention strategy and the resulting portion of deceased population. Figure 2 depicts the relation between the portion of deceases and the optimal cost of government intervention, resulting from solving the considered optimization problem (described in Eq. (16) in “Methods” section), for a range of cases for testing rate, healthcare capacity and emphasis on the acutely symptomatic and deceased population. It should be noted that all costs presented in Fig. 2 are normalised using as basis the cost of the optimal government strategy with no testing, which results to 0.01% deceases.

In particular, we considered the cases of (i) limited capacity, where two-thirds of the current healthcare capacity is used for COVID-19 patients, (ii) full capacity, where the total capacity is used and (iii) extended capacity, where the total capacity is increased by one third due to government investment and is available for COVID-19 patients. In addition, we considered the cases where no testing (Fig. 2a,d,g), slow testing (Fig. 2b,e,h) and fast testing (Fig. 2c,f,i) policies are implemented. Finally, we consider three different cases for the cost emphasis on acutely symptomatic population corresponding to no emphasis (Fig. 2a–c), low emphasis (Fig. 2d–f) and high emphasis (Fig. 2g–i). In addition, a broad range of cost weights associated with the deceased population was considered in each case, with aim to provide a rich set of strategy options. Note that a zero cost policy is only demonstrated in Fig. 2a–c since having a non-zero emphasis on the acutely symptomatic population necessarily results in an optimal intervention strategy with a non-zero cost. The exact values used to produce the results presented in Fig. 2 are provided in the “Methods” section.

From Fig. 2 we deduce the following:

(i): The healthcare system capacity significantly affects the portion of deceased population, particularly when a low/medium cost (Cost $< 40\%$) strategy with no testing is implemented. This is particularly reflected in Fig. 2a,b which demonstrates that increasing the available healthcare capacity from the limited level to the extended level results in up to a 50% decrease in deceases.
(ii): When high cost government intervention strategies are adopted (Cost > 60%), then the amount of threatened population never exceeds the healthcare capacity limit and hence its value does not affect the decease rate. The latter is demonstrated from the fact that there is no shaded regions in Fig. 2e–i.
(iii): Increasing the amount of testing enables significantly fewer deaths for the same government intervention cost. This is reflected in Fig. 2a,b, which demonstrate that slow testing approximately halves the portion of deceased population when compared to no testing, when a low intensity government strategy (Cost < 50%) is adopted. In addition, fast testing results in approximately half the deceases compared to slow testing and enables low cost strategies (Cost < 15%), as demonstrated in Fig. 2c. It should be noted though that, although fast testing policies enable a reduction in costs and decease rates, they may not always be feasible since they require sufficient resources in terms of testing equipment and trained personnel.
(iv): When a decease tolerance is set, a faster testing policy enables a less intense government strategy, and hence a lower government intervention cost. For example, when a 0.1% decease tolerance is considered, a no testing policy results in a cost of more that 90%, while slow and fast testing policies yield the same amount of deceases with costs of less than 40% and 10% respectively.

**Figure 3:**
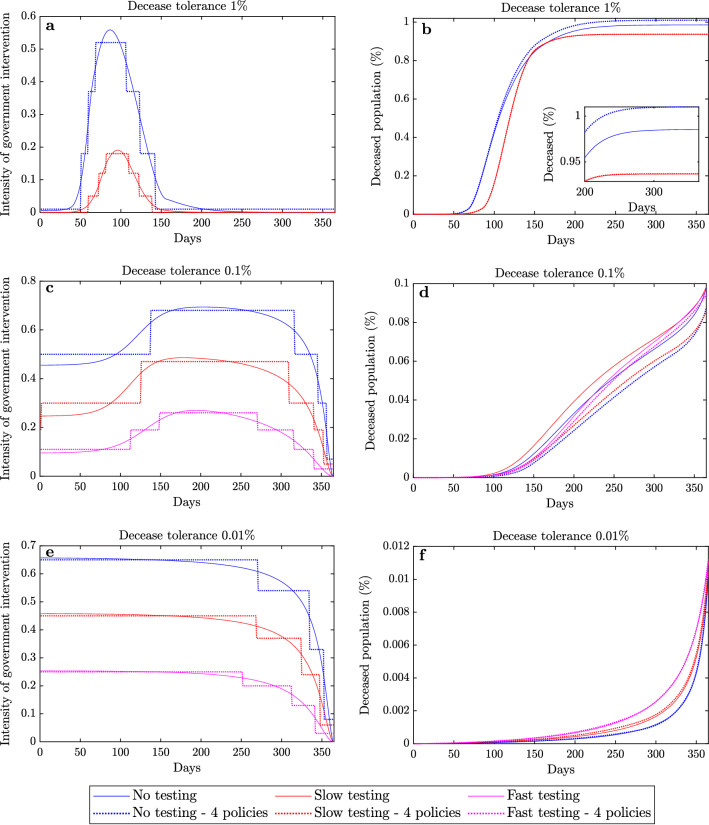
Optimal intervention strategies and deceased population. Optimal government intervention strategy (**a,c,e**) and proportion of deceased population (**b,d,f**) versus time for government strategies with 1% decease tolerance (**a,b**), 0.1% decease tolerance (**c,d**) and 0.01% decease tolerance (**e,f**) when (i) no testing (blue), (ii) slow testing (red) and (iii) fast testing (magenta) policies are adopted. The intensity of government intervention strategies corresponds to the strictness of government policies, where 0 corresponds to no interventions and 1 to the strictest possible intervention (e.g. a full scale lockdown). The approach to obtain the optimal continuous strategy is explained in the SI. Dotted plots correspond to optimized discrete implementations of the selected strategies by allowing a maximum of 4 policy levels and 6 policy changes. The distinct policy levels and the times where the policies changed were selected in an optimized way, as described in the “Methods” section and the SI (see Optimal control methodology, Algorithms 1 and 2 and Supplementary Figs. S1, S2). Implementations with 7 and 10 policy levels and 12 and 18 policy changes are presented in the SI (Supplementary Figs. S3–S10).

### Government intervention strategies

Using the findings depicted in Fig. 2, we aimed to draw efficient intervention strategies that restrict the portion of the deceased population to specific tolerated amounts with the minimum cost. The selected portions of decease tolerances were $1\%$, $0.1\%$ and $0.01\%$. The approach to obtain the optimal intervention strategies is described in the SI (Optimal control methodology). The corresponding intervention strategies for each decease tolerance level and (i) no testing, (ii) slow testing, and (iii) fast testing policy levels are depicted in Fig. 3a,c,e. Note that a fast testing policy yielded less than $1\%$ deceases for any intervention strategy. Figure 3a,c,e depicts the intensity of optimal government intervention strategies and Fig. 3b,d,f the resulting portion of deceased population for each decease tolerance level. Figure 3 demonstrates the impact of testing availability in designing intervention strategies associated with selected decease tolerances.

The intensity of government intervention is modelled in the “Methods” section with the parameter *u* (see Eqs. (9)–(15)) in the “Methods” section and Fig. 1), where a value of $u = 0$ corresponds to no government interventions and $u=1$ to the strictest intervention policy (e.g. a full scale lockdown). Note that forming actual government strategies using the provided values of *u*, is a nontrivial task. This problem is equivalent to obtaining the basic reproduction number resulting from implementing different intervention policies, which has been considered in Refs.^19–21^. For example, from Ref.^19^ it can be deduced that for Italy, a school closure policy results to $u = 0.02$ while a lockdown policy to $u = 0.8$. Motivated by this, we consider any policy with $u > 0.6$ as a *very high* intensity policy. In addition, policies with $u \in [0, 0.2]$, $u \in [0.2, 0.4]$ and $u \in [0.4, 0.6]$ are referred to as *low*, *medium* and *high* intensity policies respectively.

From Fig. 3a,b, it follows that adopting a high intensity intervention strategy ($u > 0.5$) for a period of approximately 50 days is required to limit the deceases to 1% when no testing is performed. Interestingly, a slow testing policy allows a similar portion of deceased population with a government strategy of about 3 times lower intensity. In addition, Fig. 3c,d demonstrates that a 0.1% decease tolerance requires intervention strategies ranging from high to very high $(u \in [0.5, 0.7])$, medium to high $(u \in [0.25, 0.50])$, and low to medium $(u \in [0.10, 0.25])$ intensities when no, slow and fast testing policies are adopted. Furthermore, Fig. 3e,f shows that a decease tolerance of 0.01% requires very slowly changing strategies of very high ($u \approx 0.65$), high ($u \approx 0.45$) and medium ($u \approx 0.25$) intensities when no, slow and fast testing policies are respectively adopted.

### Implementing a limited number of policies and policy changes

An implementable government strategy should only have a limited number of distinct policies. In addition, frequent changes in the intervention strategy may result in social fatigue and confusion, decreasing the receptiveness of the population to the policy instructions. The policies that follow by discretizing the continuous strategies described in the previous section are depicted in Fig. 3a,c,e with dotted plots. The approach to obtain optimized strategies with a small number of policies and policy changes is described in the SI (see Optimal control methodology, Algorithms 1 and 2 and Supplementary Figs. S1, S2). Figure 3 demonstrates that implementing 4 policies and allowing a maximum of 6 changes among them results in similar levels of decease rates compared to the continuously changing strategies. Hence, a close to optimal government response may be obtained with a relatively small number of distinct policies.

**Figure 4:**
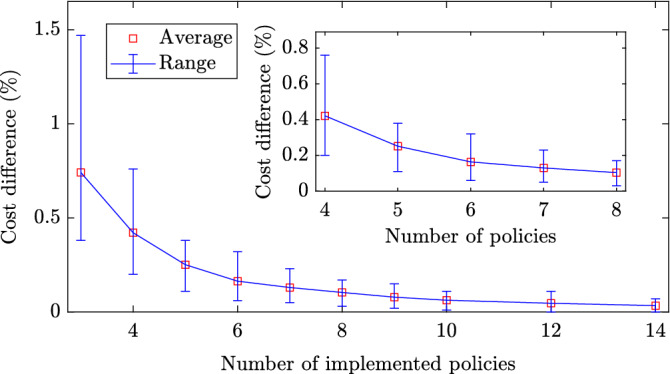
Additional cost from implementing a limited number of policies. Implementing a limited number of policies results in increased costs compared to the optimal continuously changing intervention strategy. This figure depicts the average and range of the percentage differences between the costs of the continuous strategies presented in Fig. 3 and strategies with a small number of policies, for different numbers of allowed distinct implemented policies. In each case, the number of allowed policy changes was twice the number of the implemented policies minus two. The boxed plot focuses on implementing between 4 and 8 distinct policies and demonstrates that 4 policies result in a cost difference of less than 1% in all cases.

Implementing an optimal strategy with a limited number of policies and policy changes results in an increased cost compared to the optimal continuously changing strategy. The cost differences for discrete implementations of the strategies presented in Fig. 3a,c,e are depicted in Fig. 4, where a broad range of allowed number of policies is considered. For all cases the number of allowed policy changes was twice the number of implemented policies minus two. From Fig. 4, it follows that as the number of policies grows, the percentage cost difference decreases. Furthermore, as follows from the boxed plot within Fig. 4, it can be seen that a low cost difference can be obtained with a small number of policies. In particular, adopting 4 or more intervention policies allowed a cost difference of less than 1%. The latter demonstrates the effectiveness of implementing a small number of policies and policy changes.

Hence, a small number of policies and policy changes suffices for a close to optimal government response, while at the same time resolves issues of implementability and social fatigue.

**Figure 5:**
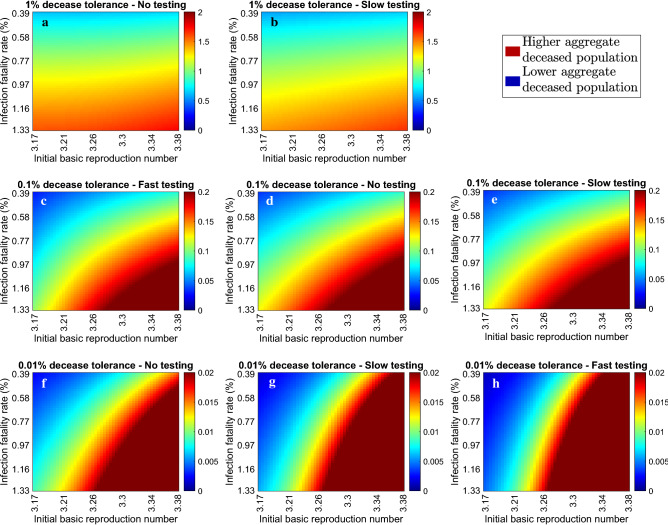
Effect of uncertainty in the initial reproduction and infection fatality rates on the aggregate deceased population. Portion of aggregate deceased population for $\bar{R}_0 \in [3.17, 3.38]$ and infection fatality rate ranging between 0.39 and 1.33% associated with decease tolerances of $1\%$ (**a,b**), $0.1\%$ (**c–e**) and $0.01\%$ (**f–h**) when no (**a,c,f**), slow (**b,d,g**), and fast (**e,h**) testing policies are implemented. Fast testing always limits the deceases to less than 1% and hence there is no corresponding case. The values were acquired by applying in each case the optimal continuous government intervention strategy obtained with $\bar{R}_0 = 3.27$ and infection fatality rate of 0.66%. Dark red and dark blue colours correspond to aggregate deceases that are at least twice the adopted tolerance levels and zero respectively. Additional results that demonstrate the impact of parametric uncertainty in forming effective government mitigation strategies are provided in the SI (Supplementary Figs. S11–S26).

**Table 1:** Effect of uncertainty in the initial reproduction and infection fatality rates—Worst case aggregate deceased population.

Decease tolerance (%)	Testing policy	Worst case aggregate deceased population (%)
1	No testing	1.74
1	Slow testing	1.65
0.1	No testing	0.29
0.1	Slow testing	0.29
0.1	Fast testing	0.28
0.01	No testing	0.057
0.01	Slow testing	0.085
0.01	Fast testing	0.116

Worst case aggregate deceases for $\bar{R}_0 \in [3.17, 3.38]$ and infection fatality rate ranging between 0.39 and 1.33% when optimal continuous intervention strategies obtained with $\bar{R}_0 = 3.27$ and infection fatality rate of $0.66\%$ are implemented. The intervention strategies are associated with decease tolerances of $1\%$, $0.1\%$ and $0.01\%$ when no, slow and fast testing policies are respectively adopted. The worst case aggregate deceased population associated with each strategy is obtained when $\bar{R}_0 = 3.38$ and the infection fatality rate is $1.33\%$, i.e. at the maximum values of the considered ranges.

### Effect of parametric uncertainty

The design of optimal control strategies relies on the use of mathematical models. Therefore, a critical aspect in designing government mitigation strategies is their dependence on parametric uncertainty, i.e. the extend of the effect of inaccurately estimating model parameters. In this section, we consider how the uncertainty in the value of the initial basic reproduction number $\bar{R}_0$ and the infection fatality rate affect the amount of deceases resulting from the strategies presented in the previous section.

In particular, we considered the effect on the aggregate deceases when the value of $\bar{R}_0$ ranges between 3.17 and 3.38 and when the infection fatality rate ranges between 0.39 and 1.33%, which correspond to $95\%$ confidence intervals, as reported in Refs.^13,16^ respectively.

Figure 5 demonstrates the effect of parametric uncertainty on the portion of deceased population resulting from each of the 8 considered government intervention strategies presented in Fig. 3a,c,e, i.e. it depicts the portion of deceased population from implementing the selected strategies when $\bar{R}_0$ and the infection fatality rate have been imprecisely estimated. Figure 5 demonstrates that the level of adopted decease tolerance is crucial when it comes to the effect of model uncertainty in the decease rate.

In particular, when a 1% decease tolerance level is adopted, the obtained strategy enabled a moderate percentage increase in the total deceases, resulting to 1.75% deceases in a worst case scenario, as demonstrated in Fig. 5a,b and Table 1. When the decease tolerance level was decreased to 0.1%, then parametric uncertainty could result in up to 0.29% deceases, i.e. about 3 times higher, as demonstrated in Fig. 5c–e and Table 1. Finally, when a decease tolerance level of 0.01% was imposed, then the considered uncertainty could result in up to 11.6 times higher values, as depicted in Fig. 5f–h and Table 1. These show that parametric uncertainty should also be taken into account in forming government policies, particularly when the adopted policies are stricter, i.e. when a low decease tolerance level is imposed.

## Discussion

Following the COVID-19 outbreak, governments across the world have adopted strict intervention policies to contain the pandemic. However, the high economic costs resulting from these policies have sparred debates^22^ on the necessity of the measures and on how these could be relaxed without risking a new wave of infections. Recently, several approaches have been proposed to control the spread of the COVID-19 pandemic. In particular, optimal intervention strategies that simultaneously minimize the number of fatalities and the economic costs are presented in Refs.^23,24^. Similar problems have been considered in Refs.^25,26^, which investigated model predictive control approaches. In addition, Ref.^27,28^ considered the problems of optimizing the quarantine and testing, and vaccination and social distancing strategies respectively. Moreover, Refs.^29–31^ explored the use of on-off policies to mitigate the effects of the pandemic, proposing control strategies that alternate between no measure and full measure policies. Furthermore, Ref.^11^ considered regional instead of national interventions to alleviate the effects of the COVID-19 pandemic. In addition, Ref.^32^ considered the problem of selecting the optimal lockdown portion that jointly minimizes the number of fatalities of the COVID-19 pandemic and the economic costs associated with the proposed policy by considering an adapted SIR model. A similar problem has been studied in Ref.^33^, which in addition considered the effect of imposing different policies to different age groups. The strategies developed to control the pandemic in China are presented in Ref.^34^. The concomitance of COVID-19 with tuberculosis and pulmonary fibrosis was considered in Refs.^35,36^ respectively.

An important missing aspect that we consider in this study is the fact that governments can only impose a limited amount of intervention policies. Such strategies offer advantages including practicality and implementability and reduce the social fatigue resulting from frequently changing policies. We demonstrate that a small number of distinct policies and policy changes yields a close to optimal government strategy. In particular, our results suggest that the additional cost incurred from implementing 4 policies and 6 policy changes is less than 1% compared to the optimal continuously changing strategy. Using tools from optimal control theory, we provide an approach, analytically described in the SI (see Optimal control methodology, Algorithms 1 and 2 and Supplementary Figs. S1, S2), which allows to select in an optimized fashion, which policies should be implemented and when should a government switch to a different policy. Our approach can be easily applied to different types of models and cost functions, which might give emphasis to other aspects of the pandemic.

A further contribution of this paper is the study of the uncertainty in the parameters associated with the initial reproduction number and the infection fatality rate. Our results suggest that parametric uncertainty has a more significant effect when stricter government policies, aiming for lower decease rates, are adopted.

This study incorporates several novel aspects. In particular, it forms an optimization problem that considers the trade-offs between the number of deceases and the social and economic costs and uses tools from optimal control theory to obtain government mitigation strategies. Moreover, it develops an algorithmic approach to produce optimized intervention strategies with a small number of policies and policy changes. Finally, it studies the impact of parametric uncertainty on the aggregate deceases resulting from optimal intervention strategies. We envision that our results will find practical applications in designing efficient intervention strategies and motivate further research on the topic.

## Methods

We consider a SIDARE model, which is a variation of the SIR model, to describe the evolution of the COVID-19 pandemic, where the population is divided in six categories: (i) Susceptible to be infected, (ii) Infected but undetected, (iii) infected and Detected, (iv) Acutely symptomatic—threatened, (v) Recovered and (vi) Extinct—deceased. Note that we use the terms threatened and acutely symptomatic, as well as deceased and extinct interchangeably.

The dynamics of the SIDARE model are given by

1 $$\begin{aligned} \dot{s} = -\beta s i, \end{aligned}$$

2 $$\begin{aligned} \dot{i} = \beta s i - \gamma _i i -\xi _i i -\nu i, \end{aligned}$$

3 $$\begin{aligned} \dot{d} = \nu i - \gamma _d d - \xi _d d, \end{aligned}$$

4 $$\begin{aligned} \dot{a} = \xi _i i + \xi _d d - \gamma _a a - \mu a, \end{aligned}$$

5 $$\begin{aligned} \dot{r} = \gamma _i i + \gamma _d d + \gamma _a a, \end{aligned}$$

6 $$\begin{aligned} \dot{e} = \mu a, \end{aligned}$$

7 $$\begin{aligned} s(0) = s_0, i(0) = i_0, d(0) = d_0, a(0) = a_0, r(0) = r_0, e(0) = e_0, \end{aligned}$$

where $s, i, d, a, r, e \in [0,1]$ are the states of the system describing the portions of susceptible, infected—undetected, infected—detected, threatened, recovered and deceased population respectively. Moreover, $s_0, i_0, d_0, a_0, r_0, e_0 \in [0, 1]$ denote the initial values for *s*, *i*, *d*, *a*, *r*, *e* respectively. The model parameters are briefly summarized below:

- $\beta$ describes the infection rate for susceptible individuals.
- $\gamma _i, \gamma _d$ and $\gamma _a$ describe the recovery rates for infected undetected, infected detected and threatened individuals.
- $\nu$ denotes the rate of detection of infected individuals, associated with the adopted level of testing.
- $\xi _i$ and $\xi _d$ describe the rates at which infected undetected and infected detected individuals become acutely symptomatic.
- $\mu$ describes the mortality rate of the disease, i.e. the rate at which acutely symptomatic individuals decease.

Note that all model parameters are assumed non-negative and constant. The SIDARE model is based on the following assumptions:

(i): Those recovered are no longer susceptible to the disease.
(ii): The considered population is constant, i.e. no births or deaths not attributed to COVID-19 are taken into account.
(iii): The considered country (or region) is isolated, i.e. no imported cases are taken into account.
(iv): Infected individuals that are detected are assumed to be quarantined, i.e. they do not contribute to new infections, something justified by existing practices.
(v): Infected individuals become acutely symptomatic before they decease.
(vi): Acutely symptomatic individuals require hospitalization since they are considered threatened for decease.

The assumption of constant population suggests that the states satisfy $s + i + d + a + r + e = 1$ at all times and hence that one state is redundant since it can be described by the remaining states at all times. In the analysis below, we select *r* to be the redundant state, satisfying $r = 1 - s - i - d - a - e$.

It should be noted that more detailed compartmental models have been proposed in the literature (e.g.^10^). Such models introduce a large number of associated parameters, resulting in possible challenges to obtain accurate estimates for their values. The level of detail of the considered SIDARE model was deemed sufficient for the purposes of this study. Simultaneously, estimates for its parameters were obtained from existing studies.

### Impact of healthcare capacity on mortality rate

An important aspect that we consider is the impact of the healthcare system capacity on the mortality rate. It is evident that when the healthcare capacity is exceeded, then the mortality rate of the population increases. The latter is modelled in Eq. (8), which suggests that the mortality rate depends on the portion of acutely symptomatic population by the relation

8 $$\begin{aligned} \overline{\mu }(a) = {\left\{ \begin{array}{ll} \mu a, \text { if } a \le \overline{h}, \\ \mu \overline{h} + \hat{\mu }(a - \overline{h}), \text { if } a > \overline{h}, \end{array}\right. } \end{aligned}$$

where the function $\overline{\mu }: [0,1] \rightarrow \mathbb {R}_+$ describes the mortality of the acutely symptomatic population. The values of $\mu$ and $\hat{\mu }$ satisfy $\mu < \hat{\mu }$ and correspond to the mortality rates when the healthcare system satisfies the demand and when the healthcare capacity is exceeded by much. This means that when the infected population increases, the mortality rate tends to $\hat{\mu }$. In addition, the value of $\overline{h}$ describes the existing healthcare capacity. Note that for simplicity we assume a constant value of $\overline{h}$, although its value could rise in the future due to a possible increase in the healthcare system capacity.

### Modelling government interventions on the SIDARE model

To account for the effect of the government actions to mitigate the spread of the pandemic, we introduce an intervention input *u* to the SIDARE model. Its value affects the infection rate of the disease, $\beta$, resulting in a slower spread. The controlled SIDARE model follows from Eqs. (1)–(7) when $\beta$ is replaced by $\beta (1-u)$ and in addition includes the healthcare capacity impact on the mortality rate, described by Eq. (8). Its dynamics are given by

9 $$\begin{aligned} \dot{s} = -\beta s i (1 - u), \end{aligned}$$

10 $$\begin{aligned} \dot{i} = \beta s i (1 - u) - \gamma _i i - \xi _i i - \nu i, \end{aligned}$$

11 $$\begin{aligned} \dot{d} = \nu i - \gamma _d d - \xi _d d, \end{aligned}$$

12 $$\begin{aligned} \dot{a} = \xi _i i + \xi _d d - \gamma _a a - \overline{\mu } (a), \end{aligned}$$

13 $$\begin{aligned} \dot{e} = \overline{\mu } (a), \end{aligned}$$

14 $$\begin{aligned} s(0) = s_0, i(0) = i_0, d(0) = d_0, a(0) = a_0, e(0) = e_0, \end{aligned}$$

15 $$\begin{aligned} s + i + d + a + r + e = 1, \end{aligned}$$

where $u \in \mathscr {U} = [0, \bar{u}]$ and $\bar{u} \le 1$ is a positive constant that denotes the maximum value that the intervention policy *u* is allowed to take. Since government actions should only aid in curtailing the effects of the pandemic, *u* is not allowed to take negative values. The dynamics of the controlled SIDARE model are depicted in Fig. 1.

The value of *u*(*t*) corresponds to the government intervention policy at time *t*, with higher values of *u* corresponding to stricter intervention policies. For example, when a government does not take any action, then $u = 0$ and when a government takes the strictest possible measures, e.g. when implementing a full scale lockdown, then $u = \bar{u}$. Note that the controlled SIDARE model (9)–(15) may describe infection waves, e.g. when model parameters change (possibly due to a new, more infectious disease variant) or when mild/no intervention policies follow a prolonged period of strict policies.

### A multi objective optimization problem

A suitable government strategy should aim to simultaneously minimize the number of fatalities and the costs associated with implementing intervention policies. The number of the aggregate fatalities during the considered period is given by *e*(*T*), where the constant $T > 0$ denotes the considered timeframe.

Moreover, any policy *u* comes with a cost associated with the social and economic side effects from its implementation. For example, a lockdown policy has an economic cost due to the inability of a portion of the population to work and a social cost associated with restricting the population movements and interactions. In addition, we consider the cost associated with the acutely symptomatic population. The latter describes the costs resulting from people requiring additional care, including possible hospitalization. These motivate the following cost functional,

$$\begin{aligned} C(u,a) = \int _{0}^{T} \frac{1}{2}u(t)^2 dt + \theta _a \int _{0}^{T} \frac{1}{2}a(t)^2 dt, \end{aligned}$$

where the non-negative parameter $\theta _a$ describes the weight given on the cost associated with the threatened population. The proposed cost functional sets a penalty analogous to the square of the intervention effort *u*, set by the government to mitigate the effects of the disease, and the square of the aggregate threatened population *a*. Note that a quadratic cost is considered in order to enable a close estimate to the non-linear cost effects arising from intense government strategies and from having a large portion of the population being in a threatened state.

However, there is a trade-off between minimizing the economic cost of government policies and the number of fatalities. The above motivates the following optimization problem

16 $$\begin{aligned} &\min _{{u(t) \in \mathscr {U}, t \in [0,T]}} J(a,e,u) \\&\text {s.t. } (9)-(15), \end{aligned}$$

where $J(a,e,u) = C(u,a) + \theta _e e(T)$ and $\theta _e$ describes the weight given to the total number of deaths in comparison with the cost associated with the threatened population and government intervention effort. The values of weight coefficients $\theta _e$ and $\theta _a$ are key to form the optimal policy. For example, if $\theta _e = \theta _a = 0$, then the focus of the government is to minimize the socio-economic cost of the intervention strategy, which trivially results to $u = 0$ for all times. On the other hand, when $\theta _a$ 7and $\theta _e$ are large, then the focus becomes to minimize the number of fatalities and the number of acutely symptomatic individuals, which results in a value of *u* that is close to $\bar{u}$ at all times. Since there is a trade-off between these objectives, selecting suitable values for $\theta _e$ and $\theta _a$ is highly important. Furthermore, note that the relative ratio between $\theta _a$ and $\theta _e$ enables an extra degree of freedom in the choice of the optimization problem and a richer set of solutions. The approach to solve the above optimization problem, using tools from optimal control theory, is explained in the SI (see Optimal control methodology).

### Implementing a limited number of policies and policy changes

A government can only implement a limited number of policies and policy changes over the time span of the pandemic, for practicality and to avoid the social fatigue resulting from frequent policy changes. To account for this, we restrict both the number of distinct implemented policies and policy changes in the previously considered optimization problem.

We denote the set of possible policies by $\mathscr {U}_d$, the set of distinct policies within strategy *u* by $\mathscr {R}(u) = \{\tilde{u}: \exists t \in [0,T] \text { s.t. } u(t) = \tilde{u}\}$ and the set of switching instants by $\mathscr {T} = \{t \in [0, T]: \lim _{\epsilon \rightarrow 0} u(t - \epsilon ) \ne \lim _{\epsilon \rightarrow 0} u(t + \epsilon )\}$. The revised problem is given by

17 $$\begin{aligned} &\min _{{u(t) \in \mathscr {U}_d, t \in [0,T]}} J(a,e,u) \\&\text {s.t. } (9)-(15), |\mathscr {R}(u)| \le \hat{n}_1, |\mathscr {T}| \le \hat{n}_2, \end{aligned}$$

where $\hat{n}_1$ and $\hat{n}_2$ denote the maximum number of policies that *u* is allowed to take from the set $\mathscr {U}_d$ and the maximum allowed number of changes in the intervention strategy over the considered timeframe respectively. The solution approach for problem (5) is analytically described within the Supplementary Information (see Optimal control methodology).

### Simulated parameters

In this section, we describe and justify the parameters considered in the simulation results presented in the “Results section. We use the controlled SIDARE model, described by Eqs. (9)–(15), for our simulations, with a time horizon of $T = 365$ days. The selected initial conditions correspond to the very early stage of the disease, where 0.001% of the population has been infected and there are no detected cases, acutely symptomatic cases, fatalities or recoveries yet. When possible, data associated with the pandemic in Italy were used for consistency.

The values of $\gamma _i$ and $\gamma _d$ were selected following^37^, which suggests a median time of disease onset to recovery for mild cases of approximately two weeks. The value of $\gamma _a$ was selected following^38^, which reported a median time between hospitalization and recovery of 12.4 days. Furthermore, to select the values for $\xi _i$ and $\xi _d$, we used the findings from Refs.^16^ on hospitalization rate per age group and data for the Italian population age distribution^39^. In addition, for the results presented in Figs. 3–5, we considered a healthcare capacity of 333 care beds per 100,000 habitants following^40^, which corresponded to the full capacity case. The maximum allowed value for *u*, given by $\bar{u}$, was selected to be 0.8 to account for the fact that complete isolation is impossible, since always some critical units will need to remain operational.

The value of $\beta$ was selected following an initial basic reproduction number of approximately 3.27 as estimated in Refs.^13^ and the relation $\overline{R}_0 = \beta s_0/ (\gamma _i + \xi _i + \nu )$ which is analytically shown in the SI (see Analysis of the SIDARE model), assuming $\nu = 0$ at $t = 0$ days. The value of $\mu$ was selected following a median infection fatality rate of 0.66%, as reported in Refs.^16^. We let $\hat{\mu }$, which corresponds to the fatality rate when the healthcare system capacity is overloaded, be 5 times higher than $\mu$, motivated by the findings in Refs.^41,42^. In addition, the values for $\theta _a$ associated with no, low and high emphasis on acutely symptomatic population were $0, 5 \times 10^4$ and $10^5$ respectively. For each case, a broad range of cost coefficients associated with the deceased population was considered, letting $\theta _e \in [0, 2.5 \times 10^4]$. The no, slow and fast testing policies corresponded to values of $\nu$ of 0, 0.05 and 0.10 respectively. Additional explanations on the simulated parameters are provided in the Supplementary Information (see Supplementary Results).

## Conclusion

We considered the problem of forming government intervention strategies that optimize the trade-off between the number of deceases and the social and economic costs. We demonstrate the relation between the number of fatalities and cost of the optimal government intervention, and how this depends on the adopted testing policy and the healthcare system capacity. Moreover, we determine that a small number of policies and policy changes suffices for a close to optimal intervention strategy. In particular, our results suggest that the additional cost incurred from implementing 4 policies and 6 policy changes is less than 1% compared to the optimal continuously changing strategy. Finally, we considered the impact of uncertainty in the initial reproduction number and infection fatality rate and demonstrated that its effect is more severe when strict government strategies, associated with lower decease tolerances, are implemented.

## Supplementary Information

Supplementary Information.

## Data Availability

All data associated with the findings of this study are available within the paper and the SI or from the corresponding author on request. The source data for all figures presented in the main text and the SI have been deposited in Zenodo (10.5281/zenodo.4433506).

## Code availability

The code to run all simulations is available at https://github.com/KIOS-Research/OptInCoV19.
