# Supplementary material for: Optimal intervention strategies to mitigate the COVID-19 pandemic effects
Source: Sci Rep. 2022 Apr 12;12:6124. doi: 10.1038/s41598-022-09857-8 (PMC9004223; doi:10.1038/s41598-022-09857-8)
Supplement: Supplementary file 1 — Supplementary Information. [file 41598_2022_9857_MOESM1_ESM.pdf]

SUPPLEMENTARY INFORMATION
Optimal intervention strategies to mitigate the COVID-19 pandemic effects

Andreas Kasis[*,a], Stelios Timotheou[a], Nima Monshizadeh[b], and Marios Polycarpou[a]

[a]Andreas Kasis, Stelios Timotheou and Marios Polycarpou are with the KIOS Research and Innovation Center of Excellence and the Department of Electrical and Computer Engineering, University of Cyprus, Cyprus; e-mails: kasis.andreas@ucy.ac.cy, timotheou.stelios@ucy.ac.cy, mpolycar@ucy.ac.cy
[b]Nima Monshizadeh is with the Engineering and Technology Institute, University of Groningen, Nijenborgh 4, 9747AG, Groningen, The Netherlands. email: n.monshizadeh@rug.nl
[*]Correspondence: kasis.andreas@ucy.ac.cy

Supplementary Information Sections

Analysis of the SIDARE model **3**

Optimal control methodology **6**

Supplementary Results **12**

Supplementary References **22**

Supplementary Figures

S1 Flowchart for Algorithm 1. 10
S2 Flowchart for Algorithm 2. 11
S3 Intervention strategy and decease rate, 1% decease tolerance, no testing 14
S4 Intervention strategy and decease rate, 1% decease tolerance, slow testing 15
S5 Intervention strategy and decease rate, 0.1% decease tolerance, no testing 15
S6 Intervention strategy and decease rate, 0.1% decease tolerance, slow testing 15
S7 Intervention strategy and decease rate, 0.1% decease tolerance, fast testing 16
S8 Intervention strategy and decease rate, 0.01% decease tolerance, no testing 16
S9 Intervention strategy and decease rate, 0.01% decease tolerance, slow testing 16
S10 Intervention strategy and decease rate, 0.01% decease tolerance, fast testing 17
S11 Effect of uncertainty in $\bar{R}_0$ on optimal strategy and decease rate, 1% decease tolerance, no testing . . 17
S12 Effect of uncertainty in $\bar{R}_0$ on optimal strategy and decease rate, 1% decease tolerance, slow testing . 17
S13 Effect of uncertainty in $\bar{R}_0$ on optimal strategy and decease rate, 0.1% decease tolerance, no testing . 18
S14 Effect of uncertainty in $\bar{R}_0$ on optimal strategy and decease rate, 0.1% decease tolerance, slow testing 18
S15 Effect of uncertainty in $\bar{R}_0$ on optimal strategy and decease rate, 0.1% decease tolerance, fast testing 18
S16 Effect of uncertainty in $\bar{R}_0$ on optimal strategy and decease rate, 0.01% decease tolerance, no testing 19
S17 Effect of uncertainty in $\bar{R}_0$ on optimal strategy and decease rate, 0.01% decease tolerance, slow testing 19
S18 Effect of uncertainty in $\bar{R}_0$ on optimal strategy and decease rate, 0.01% decease tolerance, fast testing 19
S19 Combined effect of uncertainty in $\bar{R}_0$ and infection fatality rate on the decease rate, 1% decease tolerance, no testing 20

S20 Combined effect of uncertainty in $\bar{R}_0$ and infection fatality rate on the decease rate, 1% decease tolerance, slow testing . . . 20
S21 Combined effect of uncertainty in $\bar{R}_0$ and infection fatality rate on the decease rate, 0.1% decease tolerance, no testing . . . 20
S22 Combined effect of uncertainty in $\bar{R}_0$ and infection fatality rate on the decease rate, 0.1% decease tolerance, slow testing . . . 21
S23 Combined effect of uncertainty in $\bar{R}_0$ and infection fatality rate on the decease rate, 0.1% decease tolerance, fast testing . . . 21
S24 Combined effect of uncertainty in $\bar{R}_0$ and infection fatality rate on the decease rate, 0.01% decease tolerance, no testing . . . 21
S25 Combined effect of uncertainty in $\bar{R}_0$ and infection fatality rate on the decease rate, 0.01% decease tolerance, slow testing . . . 22
S26 Combined effect of uncertainty in $\bar{R}_0$ and infection fatality rate on the decease rate, 0.01% decease tolerance, fast testing . . . 22

Supplementary Tables

S1 Model parameter values . . . 13
S2 Simulation parameters . . . 13
S3 Strategy parameters . . . 13

Preface

This Supplementary Information describes the methodology used to obtain the main results of the paper. In particular, we provide analytical results that describe the behaviour of the SIDARE model and explain how we obtain the optimal government intervention strategy. Moreover, we present a detailed algorithm that yields an optimized strategy with a limited number of policies and policy changes. Furthermore, we provide additional details regarding the numerical simulations presented in the main text and include extra simulation results with aim to enable additional clarity and intuition on the findings presented in the main text.

It should be noted that, to improve the readability and presentation of this Supplementary Information, the majority of the text in the following subsections: (i) *The SIDARE model*, (ii) *Impact of healthcare capacity on mortality rate*, (iii) *Modelling government interventions on the SIDARE model* and (iv) *A multi objective optimization problem*, is borrowed from the main text.

Notation

The set of natural numbers is denoted by $\mathbb{N}$. The projection of a scalar x to a set X is denoted by $[x]_X = \arg\min_{y\in X}|y-x|$. The interior of a set X is denoted by X^o. The cardinality of a discrete set Σ is denoted by $|\Sigma|$. A monotonically increasing (respectively decreasing) function $f:\mathbb{R}\to\mathbb{R}$ satisfies $f(x)\le f(y)$ (respectively $f(x)\ge f(y)$) for all x,y such that $x\le y$. The notation $x\ge 0$ implies that all elements of vector x are non-negative. In addition, we use $\dot{x}$ to denote the time derivative of a signal x. To avoid referring to each equation as supplementary equation, we add the prefix S in all equation labels, except those within the presented Algorithms, where the prefix A is used.

Analysis of the SIDARE model

The SIDARE model

We consider a SIDARE model, which is a variation of the SIR model, to describe the evolution of the COVID-19 pandemic, where the population is divided in six categories: (i) Susceptible to be infected, (ii) Infected but undetected, (iii) infected and Detected, (iv) Acutely symptomatic - threatened, (v) Recovered and (vi) Extinct - deceased. Note that we use the terms threatened and acutely symptomatic, as well as deceased and extinct interchangeably.

The dynamics of the SIDARE model are given by

$$\dot{s} = -\beta s i, \tag{S1a}$$

$$\dot{i} = \beta s i - \gamma_i i - \xi_i i - \nu i, \tag{S1b}$$

$$\dot{d} = \nu i - \gamma_d d - \xi_d d, \tag{S1c}$$

$$\dot{a} = \xi_i i + \xi_d d - \gamma_a a - \mu a, \tag{S1d}$$

$$\dot{r} = \gamma_i i + \gamma_d d + \gamma_a a, \tag{S1e}$$

$$\dot{e} = \mu a, \tag{S1f}$$

$$s(0) = s_0, i(0) = i_0, d(0) = d_0, a(0) = a_0, r(0) = r_0, e(0) = e_0, \tag{S1g}$$

where $s, i, d, a, r, e \in [0,1]$ are the states of the system describing the portions of susceptible, infected - undetected, infected - detected, threatened, recovered and deceased population respectively. Moreover, $s_0, i_0, d_0, a_0, r_0, e_0 \in [0,1]$ denote the initial values for s, i, d, a, r, e respectively. The model parameters are briefly summarized below:

- β describes the infection rate for susceptible individuals.
- γ_i, γ_d and γ_a describe the recovery rates for infected undetected, infected detected and threatened individuals.
- ν denotes the rate of detection of infected individuals, associated with the adopted level of testing.
- ξ_i and ξ_d describe the rates at which infected undetected and infected detected individuals become threatened.
- μ describes the mortality rate of the disease, i.e. the rate at which acutely symptomatic individuals decease.

Note that all model parameters are assumed non-negative and constant. The SIDARE model is based on the following assumptions:

- Those recovered are no longer susceptible to the disease.
- The considered population is constant, i.e. no births or deaths not attributed to COVID-19 are taken into account.
- The considered country (or region) is isolated, i.e. no imported cases are taken into account.
- Infected individuals that are detected are assumed to be isolated, i.e. they do not contribute to new infections, something justified by existing practices.
- Infected individuals become acutely symptomatic before they decease.
- Acutely symptomatic individuals are assumed to require hospitalization since they are considered threatened for decease.

The assumption of constant population suggests that the states satisfy the following condition at all times

$$s + i + d + a + r + e = 1,$$

and hence that one state is redundant since it can be described by the remaining states at all times. In the analysis below, we select r to be the redundant state, satisfying $r = 1 - s - i - d - a - e$.

Analytical results for the SIDARE model

The SIDARE model, given by equation (S1), is a bilinear system with six states. Below, we demonstrate that all states in equation (S1) take non-negative values, provided that the initial conditions are non-negative, which is clearly in line with intuition.

Lemma 1. *Consider equation* (S1) *and let all states take non-negative values at* $t = 0$*. Then, all states take non-negative values for all times.*

Proof of Lemma 1: If all states takes non-negative values at the initial conditions, then from equation (S1a), it follows that s can not take negative values by continuity and the fact that $\dot{s} = 0$ when $s = 0$. It then follows that i is also non-negative from s being non-negative and equation (S1b). Similar arguments hold for states d and a, i.e. the non-negativity of i and (i, d) respectively and the dynamics in equations (S1c)–(S1d) suffice so that d and a are non-negative. Finally, the non-negativity of (i, d, a) implies that $\dot{r} \geq 0$ and $\dot{e} \geq 0$ from equations (S1e) and (S1f) respectively and hence that r and e are also non-negative. ∎

It should also be noted that from equations (S1a), (S1e), and (S1f) and Lemma 1, it follows that the state s is monotonically decreasing and the states r and e are monotonically increasing with time.

The following lemma characterises the equilibria of equation (S1). Note that we let $x = (s, i, d, a, r, e)$ for convenience. In addition, we denote an equilibrium of (S1) by $x^* = (s^*, i^*, d^*, a^*, r^*, e^*)$.

Lemma 2. *The set of equilibria of* (S1) *is given by* $S = \{x^* : i^* = d^* = a^* = 0\}$.

Proof of Lemma 2: Any equilibrium of equation (S1) should satisfy $s^* i^* = 0$ from equation (S1a). This suggests from equation (S1b) that $i^* = 0$, which in turn suggests from equation (S1c) that $d^* = 0$. An equilibrium with $i^* = d^* = 0$ also satisfies $a^* = 0$ from equation (S1d). Moreover, consider any point in the state space of equation (S1) with $i = d = a = 0$. This point is necessarily an equilibrium since all equations (S1a)–(S1f) are zero. ∎

The following proposition enables to define the basic reproduction number $\bar{R}_0$ in terms of model parameters. Note that the definitions of stable and unstable equilibria follow from [1, Definition 4.1]. Within Proposition 1, we make use of the set $X_{\geq 0} = \{x : x \geq 0\}$.

Proposition 1. *Consider an equilibrium point of* (S1)*, given by* $x^* = (s^*, 0, 0, 0, r^*, e^*)$ *and let* $x(0)$ *take non-negative values. Then,*

(i) if $s^* < (\gamma_i + \xi_i + \nu)/\beta$*, then there exists a neighbourhood* Z *of* x^* *such that solutions initiated in* $Z \cap X_{\geq 0}$ *asymptotically converge to an equilibrium point within* Z*.*

(ii) if $s^* > (\gamma_i + \xi_i + \nu)/\beta$*, then* x^* *is an unstable equilibrium point.*

Proof of Proposition 1: We first prove part (i), using Lyapunov based arguments, and then part (ii).

Part (i): First note that since all states take non-negative values at $t = 0$, then they take non-negative values at all times from Lemma 1. Furthermore, from equations (S1a), (S1e), and (S1f) it follows that the state s is monotonically decreasing and the states r and e are monotonically increasing with time.

For the system described by equation (S1), we consider an equilibrium point x^* and the Lyapunov candidate function

$$V(x) = V_1(s, r, e) + V_2(i, d, a),$$

where

$$V_1(s, r, e) = \frac{1}{2}(s - s^*)^2 + \frac{1}{2}(r - r^*)^2 + \frac{1}{2}(e - e^*)^2,$$

and

$$V_2(i, d, a) = i + \theta_1 d + \theta_2 a,$$

where θ_1 and θ_2 are positive constants.

The dynamics of equation (S1) suggest that

$$\dot{V}_1(x) = (s - s^*)(-\beta si) + (r - r^*)(\gamma_i i + \gamma_d d + \gamma_a a) + (e - e^*)\mu a \le 0$$

where the inequality holds since $s(t) \ge s^*$, $r(t) \le r^*$ and $e(t) \le e^*$ for all times since s is monotonically decreasing and r and e are monotonically increasing and all states i, d, a and parameters $\beta, \gamma_i, \gamma_d, \gamma_d$ and μ take non-negative values.

In addition, it follows that along trajectories of equation (S1) it holds that

$$\dot{V}_2(x) = \beta si - \gamma_i i - \xi_i i - \nu i + \theta_1(\nu i - \gamma_d d - \xi_d d) + \theta_2(\xi_i i + \xi_d d - \gamma_a a - \mu a).$$

Note that, since $s^* < (\gamma_i + \xi_i + \nu)/\beta$, solutions initiated sufficiently close to x^* satisfy $s(t) < (\gamma_i + \xi_i + \nu)/\beta$ at all times due to the monotonicity of s. Therefore, letting $\rho = (\gamma_i + \xi_i + \nu) - \beta s(0)$ it follows that $\dot{i} \le -\rho i$ at all times and consequently

$$\dot{V}_2(x) \le -\rho i + \theta_1(\nu i - \gamma_d d - \xi_d d) + \theta_2(\xi_i i + \xi_d d - \gamma_a a - \mu a).$$

Hence, there exist positive θ_1, θ_2 satisfying $\theta_1 \nu + \theta_2 \xi_i < \rho$ and $\theta_2 \xi < \theta_1(\gamma_d + \xi_d)$ such that

$$\dot{V} = \dot{V}_1 + \dot{V}_2 \le -\phi_1 i - \phi_2 d - \phi_3 a \le 0, \tag{S2}$$

where ϕ_1, ϕ_2 and ϕ_3 are positive constants. Hence, there exists a compact connected set Ξ which includes x^*, given by $\Xi = \{x : V(x) \le r\}$, for sufficiently small $r > 0$, such that solutions initiated within Ξ remain in Ξ for all times. Since r can be selected to be arbitrarily small, it follows that x^* is a stable equilibrium of (S1).

LaSalle's invariance principle [1][Theorem 4.4] can now be applied on the compact and positively invariant set Ξ. This guarantees that solutions to equation (S1) initiated in Ξ converge to the largest invariant set within $\Xi \cap \{x : \dot{V}(x) = 0\}$. If $\dot{V} = 0$ holds within Ξ it follows from equation (S2) that $(i, d, a) = (0, 0, 0)$. The latter implies convergence of the states (s, r, e) from equations (S1a) and (S1e)–(S1f) to the set of equilibria within Ξ.

In addition, note that if r in the definition of Ξ is selected to be sufficiently small, then Ξ contains only stable equilibria, satisfying $s^* < (\gamma_i + \xi_i + \nu)/\beta$. This allows to deduce that solutions initiated within Ξ asymptotically converge to an equilibrium point by using arguments analogous to those in [2, Prop. 4.7, Theorem 4.20].

Part (ii): Consider any equilibrium x^* such that $s^* > (\gamma_i + \xi_i + \nu)/\beta$. Then, the Jacobian matrix associated with the linearisation of equation (S1) at x^* is given by

$$J_{x^*} = \begin{bmatrix} 0 & -\beta s^* & 0 & 0 & 0 & 0 \\ 0 & \beta s^* - \gamma_i - \xi_i - \nu & 0 & 0 & 0 & 0 \\ 0 & \nu & -\gamma_d - \xi_d & 0 & 0 & 0 \\ 0 & \xi_i & \xi_d & -\gamma_a - \mu & 0 & 0 \\ 0 & \gamma_i & \gamma_d & \gamma_a & 0 & 0 \\ 0 & 0 & 0 & \mu & 0 & 0 \end{bmatrix}.$$

It can easily be shown that J_{x^*} has three zero eigenvalues and three more given by $-\gamma_d - \xi_d$, $-\gamma_a - \mu$ and $\beta s^* - \gamma_i - \xi_i - \nu$. When $s^* > (\gamma_i + \xi_i + \nu)/\beta$, the last eigenvalue is positive which suggests that the linearisation of (S1) at x^* is unstable. ■

The value of $\bar{R}_0$ corresponds to the basic reproduction rate. Stability of the dynamics described by equation (S1) imply a basic reproduction rate of less than or equal to 1. Hence, for the considered case it holds that $\bar{R}_0 \bar{s} = 1$. Therefore, from Proposition 1 it follows that the value of $\bar{R}_0$ is given by $\bar{R}_0 = 1/\bar{s} = \beta/(\gamma_i + \xi_i + \nu)$.

Impact of healthcare capacity on mortality rate

An important aspect that we consider is the impact of the healthcare system capacity on the mortality rate. It is evident that when the healthcare capacity is exceeded, then the mortality rate of the population increases. The latter is modelled in equation (S3), which suggests that the mortality rate depends on the portion of acutely symptomatic population by the relation

$$\overline{\mu}(a) = \begin{cases} \mu a, & \text{if } a \le \overline{h}, \\ \mu \overline{h} + \hat{\mu}(a - \overline{h}), & \text{if } a > \overline{h}, \end{cases} \tag{S3}$$

where the function $\overline{\mu} : [0,1] \to \mathbb{R}$ describes the mortality of the acutely symptomatic population. The values of μ and $\hat{\mu}$ satisfy $\mu < \hat{\mu}$ and correspond to the mortality rates when the healthcare system satisfies the demand and when the healthcare capacity is exceeded by much. This means that when the infected population increases, the mortality rate tends to $\hat{\mu}$. In addition, the value of $\overline{h}$ describes the existing healthcare capacity. Note that for simplicity we assume a constant value of $\overline{h}$, although its value could rise in the future due to a possible increase in the healthcare system capacity.

It should be noted that all all analytical results presented above, can be trivially extended when the term μa in equations (S1d) and (S1f) is replaced with $\overline{\mu}(a)$.

Modelling government interventions on the SIDARE model

To account for the effect of the government actions to mitigate the spread of the pandemic, we introduce an intervention input u to the SIDARE model, described by equation (S1). Its value affects the infection rate of the disease, β, resulting in a slower spread. The controlled SIDARE model follows from equation (S1) when β is replaced by $\beta(1-u)$ and in addition includes the healthcare capacity impact on the mortality rate, described by equation (S3). Its dynamics are given by

$$\dot{s} = -\beta s i(1-u), \tag{S4a}$$
$$\dot{i} = \beta s i(1-u) - \gamma_i i - \xi_i i - \nu i, \tag{S4b}$$
$$\dot{d} = \nu i - \gamma_d d - \xi_d d, \tag{S4c}$$
$$\dot{a} = \xi_i i + \xi_d d - \gamma_a a - \overline{\mu}(a), \tag{S4d}$$
$$\dot{e} = \overline{\mu}(a), \tag{S4e}$$
$$s(0) = s_0, i(0) = i_0, d(0) = d_0, a(0) = a_0, e(0) = e_0, \tag{S4f}$$
$$s + i + d + a + r + e = 1, \tag{S4g}$$

where $u \in \mathcal{U} = [0, \bar{u}]$ and $\bar{u}$ is a positive constant that denotes the maximum value that the intervention policy u is allowed to take. Since government actions should only aid in curtailing the effects of the pandemic, $u(t)$ is not allowed to take negative values. It is also assumed that $\bar{u} < 1$ to account for the fact that complete isolation is impossible, since always some critical units will need to remain operational.

The value of $u(t)$ corresponds to the government intervention policy at time t, with higher values of u corresponding to stricter intervention policies. For example, when a government does not take any action, then $u = 0$ and when a government takes the strictest possible measures, e.g. when implementing a full scale lockdown, then $u = \bar{u}$.

Optimal control methodology

A multi objective optimization problem

A suitable government strategy should aim to simultaneously minimize the number of fatalities and the costs associated with implementing intervention policies. The number of the aggregate fatalities during the considered period, is described by the term $e(T)$, where the constant $T > 0$ denotes the considered timeframe.

Moreover, any policy u comes with a cost associated with the social and economic side effects from its implementation. For example, a lockdown policy has an economic cost due to the inability of a portion of the population to work and a social cost associated with restricting the population movements and interactions. In addition, we consider the cost associated with the acutely symptomatic population. The latter describes the costs resulting from

people requiring additional care, including possible hospitalization. These motivate the following cost functional,

$$C(u,a) = \int_0^T \frac{1}{2}u(t)^2 dt + \theta_a \int_0^T \frac{1}{2}a(t)^2 dt,$$

where the non-negative parameter θ_a describes the weight given on the cost associated with the threatened population. The proposed cost functional sets a penalty analogous to the square of the intervention effort u and set by the government to mitigate the effects of the disease and the square of the aggregate infected population a. Note that a quadratic cost is considered in order to enable a close estimate to the non-linear cost effects arising from intense government strategies and from having a large portion of the population being in a threatened state.

However, there is a trade-off between minimizing the economic cost of government policies and the number of fatalities. The above motivates the following combined cost functional

$$J(a,e,u) = C(u,a) + \theta_e e(T),$$

where θ_e describes the weight given to the total number of deaths in comparison with the cost associated with the threatened population and government intervention effort. The values of weight coefficients θ_e and θ_a are key to form the optimal policy. For example, if $\theta_e = \theta_a = 0$, then the focus of the government is to minimize the cost of the intervention strategy, which trivially results to $u(t) = 0$ for all times. On the other hand, when θ_a and θ_e are large, then the focus becomes to minimize the number of fatalities and the number of acutely symptomatic individuals, which results in a value of u that is close to $\bar{u}$ at all times. Since there is a trade-off between these objectives, selecting suitable values for θ_e and θ_a is highly important. Furthermore, note that the relative ratio between θ_a and θ_e enables an extra degree of freedom in the choice of the optimization problem and a richer set of solutions.

The above enable to define the following optimization problem, which is considered in the following section:

$$\begin{aligned} &\min_{u(t)\in\mathcal{U},t\in[0,T]} J(a,e,u) \\ &\text{s.t. (S4).} \end{aligned} \tag{S5}$$

Furthermore, noting that the states a and e are uniquely defined by the initial conditions and u, then a functional $\mathcal{J}$ can be defined such that $\mathcal{J}(u) = J(a,e,u)$ for given initial conditions. Hence, the optimization problem Problem (S5) can be equivalently written as

$$\begin{aligned} &\min_{u(t)\in\mathcal{U},t\in[0,T]} \mathcal{J}(u) \\ &\text{s.t. (S4).} \end{aligned} \tag{S6}$$

Solution approach

We call $\hat{u}$ a solution to Problem (S5) if $\mathcal{J}(\hat{u}) = \min_{u(t)\in\mathcal{U},t\in[0,T]} \mathcal{J}(u) = \min_{u(t)\in\mathcal{U},t\in[0,T]} J(a,e,u)$ subject to (S4). We call the solution $\hat{u}$ and the corresponding state $\hat{x}$ an optimal pair. The existence of a solution to Problem (S5) is established by the following lemma.

Lemma 3. *There exists a solution to Problem (S5).*

Proof of Lemma 3: To prove Lemma 3, we apply [4, Thm. 2.1, p. 63], which provides sufficient conditions for the existence of an optimal solution to a general optimal control problem. In particular, the conditions in [4, Thm. 2.1, p. 63] are satisfied for Problem (S5) since:

(i) Equation (S4) is continuously differentiable,

(ii) there exist a feasible solution to equation (S4),

(iii) $\mathcal{U}$ is a compact set,

(iv) $F(t,x) = \{[-\beta si(1-u)\ ,\ \beta si(1-u) - \gamma_i i - \xi_i i - \nu i\ ,\ \nu i - \gamma_d d - \xi_d d\ ,\ \xi_i i + \xi_d d - \gamma_a a - \overline{\mu}(a)\ ,\ \overline{\mu}(a)]^T : u \in \mathcal{U}\}$ is convex for all $(t,s,i,d,a,e) \in [0,T]\times[0,1]^5$.

Hence, there exists an optimal solution $\hat{u}$ to Problem (S5). ■

To obtain the optimal intervention strategy $\hat{u}$, it will be convenient to form the Hamiltonian for Problem (S5), as below

$$H(x,u,\lambda) = \frac{1}{2}u^2 + \frac{1}{2}\theta_a a^2 + \lambda^T(f_0(x) + f_1(x)u), \tag{S7}$$

where $\lambda \in \mathbb{R}^5$ is called the co-state of the system and $f_0(x)$ and $f_1(x)$ follow from equation (S4) and are given[1] by

$$f_0(x) = \begin{bmatrix} -\beta s i \\ \beta s i - \gamma_i i - \xi_i i - \nu i \\ \nu i - \gamma_d d - \xi_d d \\ \xi_i i + \xi_d d - \gamma_a a - \overline{\mu}(a) \\ \overline{\mu}(a) \end{bmatrix}, \; f_1(x) = \begin{bmatrix} \beta s i \\ -\beta s i \\ 0 \\ 0 \\ 0 \end{bmatrix}.$$

Below, we provide necessary optimality conditions for Problem (S5), which are a result of Pontryagin's minimum principle.

Proposition 2. *Let $(\hat{x}, \hat{u})$ be a locally optimal pair to Problem (S5). Then, there exists a co-state function $\hat{\lambda} : [0,T] \to \mathbb{R}^5$ such that the following conditions hold for almost all $t \in [0,T]$:*

$$\dot{\hat{\lambda}}^T = -(\hat{\lambda}^T(\nabla f_0(\hat{x}) + \nabla f_1(\hat{x})\hat{u}) + \frac{1}{2}\theta_a \nabla a^2), \tag{S8a}$$

$$\hat{\lambda}(T) = [0\;0\;0\;0\;\theta_e]^T, \tag{S8b}$$

$$\hat{u} = [-\hat{\lambda}^T f_1(\hat{x})]_{\mathcal{U}} \tag{S8c}$$

Proof of Proposition 2: The proof follows from applying Pontryagin's minimum principle [5] in Problem (S5). The principle states that for a trajectory $(\hat{x}, \hat{u})$ that solves Problem (S5), there exists a function $\hat{\lambda} : [0,T] \to \mathbb{R}^5$ such that:

$$\dot{\hat{x}}^T = \frac{\partial H}{\partial \lambda}(\hat{x}(t), \hat{u}(t), \hat{\lambda}(t)), \; \hat{x}(0) = x_0, \tag{S9a}$$

$$\dot{\hat{\lambda}}^T = -\frac{\partial H}{\partial x}(\hat{x}(t), \hat{u}(t), \hat{\lambda}(t)), \tag{S9b}$$

$$\hat{\lambda}(T) = \frac{\partial S}{\partial x}(\hat{x}(T))^T, \tag{S9c}$$

$$H(\hat{x}(t), \hat{u}(t), \hat{\lambda}(t)) \le H(\hat{x}(t), u(t), \hat{\lambda}(t)), \quad \forall u \in \mathcal{U}, \tag{S9d}$$

where the Hamiltonian H is given by equation (S7) and S represents the final cost, given by $S(x) = \theta_e e$. Hence, equation (S8) follows directly[2] from equations (S7) and (S9). Note that equation (S8c) follows from equation (S9d), which results to $\frac{\partial H}{\partial u}(\hat{x}(t), \hat{u}(t), \hat{\lambda}(t)) = 0$ when $\hat{u}(t) \in \mathcal{U}^\circ$, i.e. when $\hat{u}(t)$ lies in the interior of $\mathcal{U}$. ■

We make use of equation (S8) and the controlled SIDARE model dynamics, described by equation (S4) to obtain the optimal solution to Problem (S5). To tackle the challenges associated with dealing with initial and final value constraints in numerical simulations, we used an adapted forward-backward sweep method (e.g. [8, Ch. 21]).

Implementing a limited amount of policies and policy changes

An implementable government strategy requires that u takes a small number of distinct values, i.e. there exists a finite set of distinct possible intervention policies $\mathcal{U}_d = \{\tilde{u}_1, \tilde{u}_2, \ldots, \tilde{u}_{\tilde{m}}\} \subset \mathcal{U}$ such that $u(t) \in \mathcal{U}_d$ for all $t \ge 0$. For notational convenience, it is assumed that $\tilde{u}_i < \tilde{u}_j$ for $i < j$. Moreover, for each strategy u, we consider the set of policy change instants $\mathcal{T} = \{t_1, t_2, \ldots t_n\}$ satisfying $0 < t_1 < t_2 < \cdots < t_n < T$, where $T > 0$ denotes the considered timeframe, such that $u(t) = u_j \in \mathcal{U}_d, t \in [t_{j-1}, t_j), 1 \le j \le n+1$, where $t_0 = 0$ and $t_{n+1} = T$. In addition, it is assumed that $u_j \ne u_{j+1}, 1 \le j \le n-1$. Note that the cardinality of the set $\mathcal{T}$ is important as it describes the amount of changes between policies. For any intervention strategy u, we let $\mathcal{R}(u) = \{\tilde{u} : \exists t \in [0,T] \text{ s.t. } u(t) = \tilde{u}\}$ satisfying $\mathcal{R}(u) \subseteq \mathcal{U}_d$ denote the set of policies within u. In addition, $|\mathcal{R}(u)|$ describes the number of distinct policies within strategy u.

Drawing a practical government intervention strategy motivates the introduction of two constraints: (i) on the number of distinct policies, so that a small set of rules is implemented by the population, (ii) on the number of policy

[1] Note that the state r within x does not appear in either $f_0(x)$ or $f_1(x)$. We defined f_0 and f_1 as functions of x to avoid introducing extra notation.

[2] Note that there is a technical issue in the definition of $\frac{\partial H}{\partial x}$ when $a = \bar{h}$ since $\frac{d\bar{\mu}(a)}{da}$ is not well defined at this point. This issue can be resolved by considering the subdifferential of $\bar{\mu}(a)$ at $a = \bar{h}$ [6], and define the solutions for x and λ in the sense of Filippov [7]. We refrain from properly defining these concepts to avoid the introduction of extensive technical notions and to keep the focus of the paper on the practical aspects of the results.

changes, since frequent policy changes may result in social fatigue, decreasing the responsiveness of the population to the policy instructions. To account for these, we define the following optimization problem:

$$\begin{aligned} &\min_{u(t)\in\mathcal{U}_d, t\in[0,T]} J(a,e,u) \\ &\text{s.t. } (\text{S4}), |\mathcal{R}(u)| \leq \hat{n}_1, |\mathcal{T}| \leq \hat{n}_2, \end{aligned} \tag{S10}$$

where $\hat{n}_1$ and $\hat{n}_2$ respectively denote the maximum number of policies that u is allowed to take from the set $\mathcal{U}_d$ and the maximum allowed number of changes in the intervention policy over the considered timeframe. In analogy to (S6), Problem (S10) can be equivalently written as

$$\begin{aligned} &\min_{u(t)\in\mathcal{U}_d, t\in[0,T]} \mathcal{J}(u) \\ &\text{s.t. } (\text{S4}), |\mathcal{R}(u)| \leq \hat{n}_1, |\mathcal{T}| \leq \hat{n}_2. \end{aligned} \tag{S11}$$

To solve the above problem, we make use of the solution to Problem (S5), which is a continuous relaxation to Problem (S10), and hence provides a lower bound to the cost of Problem (S10). Our approach to obtain a solution to Problem (S10) is described below:

(i) Obtain $\hat{u}$ that solves Problem (S5).

(ii) Using $\hat{u}$, obtain an optimized solution to Problem (S10), denoted by $\hat{u}_d$, resulting from a local minimum cost search algorithm.

Part (i) of the above approach is explained in the "Solution approach" section above (see also Proposition 2). Part (ii) is described in detail below.

Part (ii): The methodology in this section is split in two steps. First, we obtain a strategy that approximates $\hat{u}$ and simultaneously satisfies the constraints of Problem (S10). Using this strategy for initialization, we implement Algorithm 1 which produces the optimized policy $\hat{u}_d$. Note the in the presented process, it is assumed that $\hat{n}_1 > 1$ and $\hat{n}_2 \geq 1$. The case where $\hat{n}_1 = 1$ which necessarily results in $|\mathcal{T}| = \emptyset$ can be trivially solved by evaluating the costs of implementing each value of $u \in \mathcal{U}_d$.

Initialization: The first step aims to obtain an intervention strategy $\bar{u}_d$ that approximates the continuous strategy $\hat{u}$ and simultaneously satisfies the constraints of the problem. To obtain such strategy, we let $u_{max} = \max_{t\in[0,T]} \hat{u}$ and $u_{min} = \min_{t\in[0,T]} \hat{u}$ and define $\hat{u}_i = [u_{min} + (i-1) * (u_{max} - u_{min})/(\hat{n}_1 - 1)]_{\mathcal{U}_d}$ and $U_s = \{\hat{u}_i : i \in \{1,\dots,\hat{n}_1\}\}$, where $\hat{n}_1$ is given in Problem (S10). Consequently, we let $v_d(t) = \arg\min_{y\in U_s} |y - \hat{u}(t)|$ for all $t \in [0,T]$, i.e. we project the values of the continuous policy $\hat{u}$ onto the discrete set U_s. The latter leads to $\mathcal{R}(v_d) = U_s$. Moreover, we define the set $\bar{\mathcal{T}}^i = \{t \in [0,T] : \lim_{\epsilon\to 0} v_d(t+\epsilon) \neq \lim_{\epsilon\to 0} v_d(t-\epsilon)\}$, i.e. the set of all time instants when a change occurs in the intervention policy v_d.

If $|\bar{\mathcal{T}}^i| \leq \hat{n}_2$, then we select $\bar{u}_d = v_d$. Otherwise, we define $|\bar{\mathcal{T}}^i|$ strategies $v_{d,j}$, such that strategy $v_{d,j}$ satisfies $v_{d,j}(t) = v_d(t), t \in [0,T] \setminus [t_j, t_{j+1})$ and $v_{d,j}(t) = v_d(t_{j-1}), t \in [t_j, t_{j+1})$, i.e. $v_{d,j}$ follows by omitting the jth switch in v_d. We then calculate the cost for each of the above policies, given by $\mathcal{J}(v_{d,j})$, and select the $q = |\bar{\mathcal{T}}^i| - \hat{n}_2$ strategies with the lowest cost. Finally, we neglect the switches associated with these q strategies from v_d, i.e. the q switches which individually cause the least increase in cost once removed, to construct $\bar{u}_d$. The latter enables the construction of $\bar{u}_d$ which satisfies all the constraints of Problem (S10).

Algorithm 1: After obtaining $\bar{u}_d$, as explained above, then an optimized solution $\hat{u}_d$ is obtained from Algorithm 1, as described below. To initialise Algorithm 1, we first consider the set of switching instants for strategy $\bar{u}_d$, which we denote by $\bar{\mathcal{T}}$, and select a sufficiently small $\delta > 0$ which corresponds to the tolerance of Algorithm 2, implemented within Algorithm 1. In addition, the vector p, with $p \in \mathbb{N}^{|\mathcal{R}(\bar{u}_d)|}$, represents the indices of implemented policies from $\mathcal{U}_d$ in strategy $\bar{u}_d$ in decreasing order, i.e. $p_i = k$ means that the ith largest implemented policy is $\tilde{u}_k$, reminding that $\mathcal{U}_d = \{\tilde{u}_1, \tilde{u}_2, \dots, \tilde{u}_{\bar{m}}\}$.

Algorithm 1 aims to obtain an optimized intervention strategy, denoted by $\hat{u}_d$, by making incremental changes in the test policy $\hat{u}_t$, as seen in equations (A.1.1)–(A.1.2), e.g. when $m = 1$ and $n = 1$ then Algorithm 1 replaces the largest policy within $\hat{u}_t$ with the immediately more relaxed policy within $\mathcal{U}_d$, in analogy when $n = 2$ then the attempted policy is the one which is the immediately more strict. Then, Algorithm 2, described in detail below, makes use of the test policy $\hat{u}_t$ and set of switching times $\mathcal{T}$ and provides a new test policy with an optimized set of switching times, denoted by $\tilde{\mathcal{T}}$, and a corresponding cost, denoted by C. If C is less than the lowest cost obtained by that stage, denoted by C_m, then $\hat{u}_d$ is updated as in equation (A.1.4.1). In addition, the supplementary variables C_m, p and $\bar{\mathcal{T}}$ are updated to facilitate the progression of the algorithm, as described in equations (A.1.4.2)–(A.1.4.4). Moreover, the convergence variable θ takes the value of 0, which imposes that a new set of iterations need to be performed for the convergence of the algorithm. If there is no improvement in C_m for all $m \in \{1,\dots,|\mathcal{R}(\hat{u}_d)|\}$ and

$n \in \{1, 2\}$, then Algorithm 1 converges. Convergence of Algorithm 1 suggests that any incremental change in any of the policies within $\hat{u}_t$ will result in a higher cost, and hence that a local minimum has been reached. For improved clarity, a flowchart of Algorithm 1 is presented in Supplementary Fig. S1.

Algorithm 2: Algorithm 2 aims to obtain an optimized set of switching times, denoted by $\tilde{\mathcal{T}}$ and a corresponding intervention strategy $\hat{u}_t$ by making use of the discrete test policy $\hat{u}_t$, the set of switching times $\mathcal{T}$ and the tolerance level δ provided from Algorithm 1 for its initialization. In particular, it alters the switching times by δ in either direction, as demonstrated in equation (A.2.1) and updates $\bar{u}_t$ as described in equations (A.2.2)–(A.2.3). Then, a new cost is calculated, based on the new strategy $\bar{u}_t$ by implementing equation (A.2.4). If the new cost, denoted by $\tilde{C}$ is lower than the cost obtained by policy $\hat{u}_t$, then the cost, the policy and the set of switching times are updated, as demonstrated in equations (A.2.5.1)–(A.2.5.3). Otherwise, the policy $\bar{u}_t$ reverts to its previous value. In addition, the variable ϕ serves as a convergence variable. In particular, when any iteration occurs that allows a decrease in cost then its value is set to 0, which results in a new set of iterations for $\tilde{\mathcal{T}}$. Convergence of the algorithm suggests that a local minimum of the cost C is reached, where changing any switching time in $\tilde{\mathcal{T}}$ does not result in a lower cost. For improved intuition, Supplementary Fig. S2 depicts a flowchart of Algorithm 2.

Algorithm 1 creates a monotonically decreasing sequence of values for C, which is lower bounded by $C^* = \mathcal{J}(\hat{u})$, i.e. the cost associated with the optimal continuous policy obtained in part (i). Therefore, the sequence of updates in C and hence Algorithm 1 converge, as directly follows by the monotone convergence theorem (e.g. [9, Theorem 2.4.2]). A similar argument follows for the convergence of Algorithm 2.

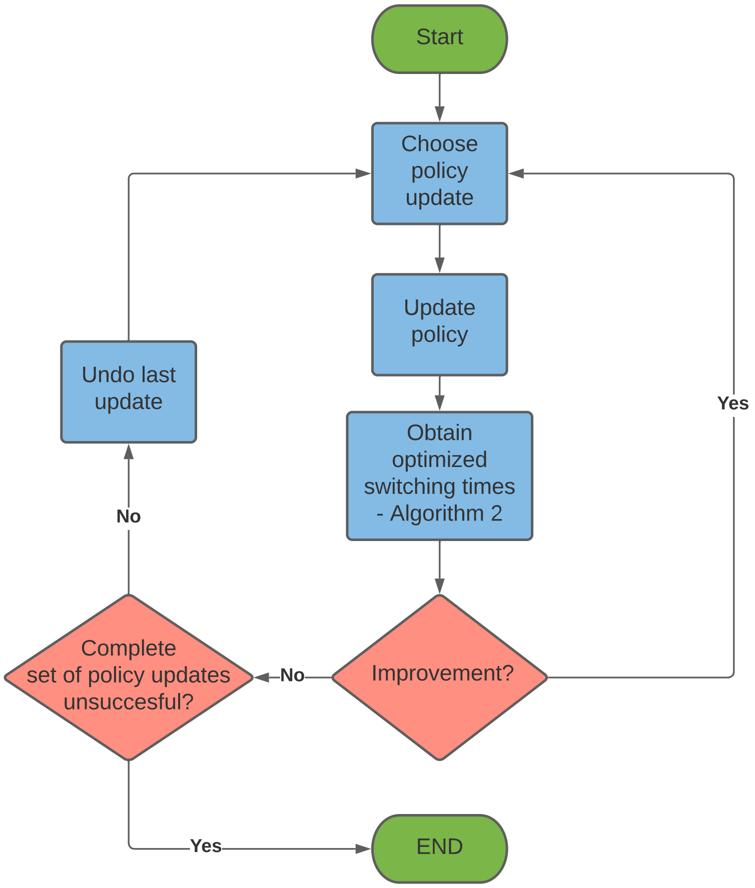

Supplementary Figure S1: Flowchart for Algorithm 1.

Algorithm 1: Scheme to obtain $\hat{u}_d$.

Inputs: $\bar{\mathcal{T}}, \bar{u}_d, \delta, \mathcal{U}_d, p$.

Output: $\hat{u}_d$.

Initialization:

$C = \mathcal{J}(\bar{u}_d), C_m = C, \mathcal{T} = \bar{\mathcal{T}}, \hat{u}_d = \hat{u}_t = \bar{u}_d, \theta = 0.$

while $\theta = 0$ **do**

$\theta = 1,$

for $m = 1 : |\mathcal{R}(\hat{u}_d)|$

for $n = 1 : 2$

$$\hat{\mathcal{T}} = \{t : \hat{u}_d(t) = \tilde{u}_{p_m}\}, \tag{A.1.1}$$

$$\hat{u}_t(t) = \tilde{u}_{[p_m + (-1)^n]_{[1,\bar{m}]}}, t \in \hat{\mathcal{T}}, \tag{A.1.2}$$

$$[\tilde{\mathcal{T}}, \hat{u}_t, C] = \text{Algorithm } 2(\mathcal{T}, \hat{u}_t, \delta) \tag{A.1.3}$$

if $C < C_m$,

$$\hat{u}_d = \hat{u}_t, \tag{A.1.4.1}$$

$$C_m = C, \tag{A.1.4.2}$$

$$p_m = [p_m + (-1)^n]_{[1,\bar{m}]}, \tag{A.1.4.3}$$

$$\mathcal{T} = \tilde{\mathcal{T}}, \tag{A.1.4.4}$$

$$\theta = 0, \tag{A.1.4.5}$$

else

$$\hat{u}_t = \hat{u}_d, \tag{A.1.4.6}$$

end

end

end

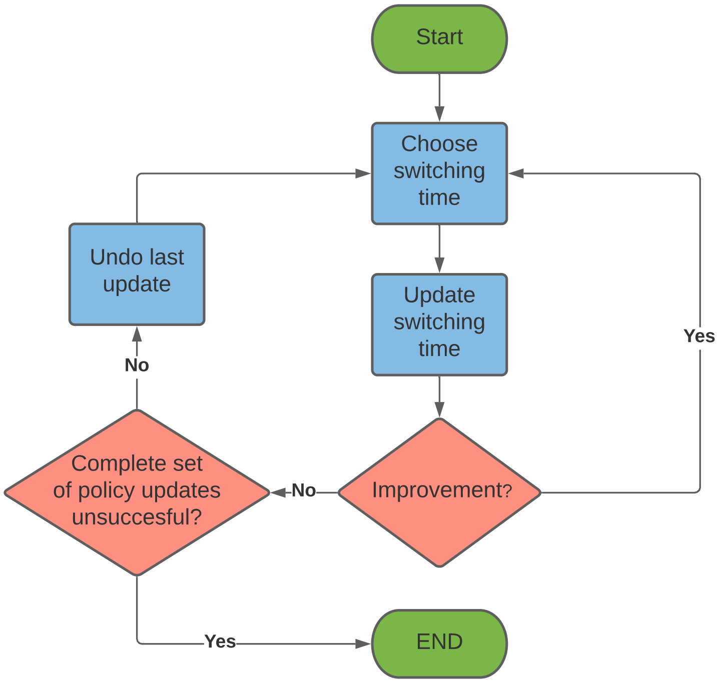

Supplementary Figure S2: Flowchart for Algorithm 2.

Algorithm 2: Scheme to obtain $\tilde{\mathcal{T}}, \hat{u}_t, C$.

Inputs: $\mathcal{T}, \hat{u}_t, \delta$.
Output: $\tilde{\mathcal{T}}_i, 1 \le i \le |\mathcal{T}|, \hat{u}_t, C$.
Initialization:
$C = \mathcal{J}(\hat{u}_t), \bar{u}_t = \hat{u}_t, \tilde{\mathcal{T}} = \mathcal{T}, \phi = 0, \tilde{\mathcal{T}}_0 = 0, \tilde{\mathcal{T}}_{|\mathcal{T}|+1} = T.$
while $\phi = 0$ **do**
  $\phi = 1$,
  for $k = 1 : |\mathcal{T}|$
    for $j = 1 : 2$

$$\hat{\delta} = \delta \times (-1)^j, \tag{A.2.1}$$

$$\tilde{\mathcal{S}} = \begin{cases} [[\tilde{\mathcal{T}}_k + \hat{\delta}]_{[\tilde{\mathcal{T}}_{k-1}, \tilde{\mathcal{T}}_k]}, \tilde{\mathcal{T}}_k], & \text{if } j = 1, \\ [\tilde{\mathcal{T}}_k, [\tilde{\mathcal{T}}_k + \hat{\delta}]_{[\tilde{\mathcal{T}}_k, \tilde{\mathcal{T}}_{k+1}]}], & \text{if } j = 2, \end{cases} \tag{A.2.2}$$

$$\bar{u}_t(t) = \hat{u}_t(\tilde{\mathcal{T}}_{k+1-j}), \forall t \in \tilde{\mathcal{S}}, \tag{A.2.3}$$

$$\tilde{C} = \mathcal{J}(\bar{u}_t), \tag{A.2.4}$$

    if $\tilde{C} < C$

$$C = \tilde{C}, \tag{A.2.5.1}$$

$$\hat{u}_t = \bar{u}_t, \tag{A.2.5.2}$$

$$\phi = 0, \tag{A.2.5.3}$$

$$\tilde{\mathcal{T}}_k = [\tilde{\mathcal{T}}_k + \hat{\delta}]_{[\tilde{\mathcal{T}}_{k-1}, \tilde{\mathcal{T}}_{k+1}]}, \tag{A.2.5.4}$$

    else

$$\bar{u}_t = \hat{u}_t, \tag{A.2.5.5}$$

    end

    end
  end

Supplementary Results

In this section, we present additional results that supplement the findings presented in the main text. In particular, we present optimized strategies and the corresponding decease rates when 4, 7 and 10 distinct policies and 6, 12 and 18 policy changes are allowed, as opposed to only the case of 4 distinct policies and 6 policy changes that is presented in the main text. Furthermore, we complement our results associated with the effect of parametric uncertainty, depicted in Fig. 5 of the main text, with additional results demonstrating the optimal strategies and corresponding aggregate deceases for a range of values for the infection fatality rate and initial basic reproduction number $\bar{R}_0$. These results aim to provide additional intuition on the effect of parametric uncertainty in forming efficient government strategies.

Simulated parameters

The controlled SIDARE model, described by equation (S4), has been used in our simulations with a time horizon of $T = 365$ days. The selected initial conditions correspond to the very early stage of the disease, where 0.001% of the population has been infected and there are no detected cases, acutely symptomatic cases, fatalities or recoveries yet.

The values of γ_i and γ_d were selected following [10], which suggests a median time of disease onset to recovery for mild cases of approximately two weeks. The value of γ_a was selected following [11], which reported a median time between hospitalization and recovery of 12.4 days.

The value of β was selected to be 0.251, corresponding to an initial basic reproduction number $\overline{R}_0 = \beta s_0/(\gamma_i + \xi_i + \nu)$ of approximately 3.27 following [12], and assuming $\nu = 0$ at $t = 0$ days. The value of $\bar{u}$, corresponding to the maximum allowed value for the input u was selected to be 0.8. Furthermore, to select the values for ξ_i and ξ_d for the case of Italy, we used the findings from [13] on hospitalization rate per age group and data for the Italian population age distribution [14]. In addition, we considered 333 care beds per 100,000 habitants following [15].

The value of μ was selected to be 0.0085, being associated with an infection fatality rate of 0.66% as reported in [13]. The latter is in agreement with various studies that report a mortality rate close or below 1% [16], [17]. We let $\hat{\mu}$, which corresponds to the fatality rate when the healthcare system capacity is overloaded, be 5 times higher than μ, motivated from [18] which compared the fatality rates in two regions in Italy, Lombardy where more than 80% of the healthcare capacity was held by COVID-19 patients and Veneto where up to 40% was held, and deduced an approximate five fold increase in the mortality rate.

To facilitate the reproducibility of the main results, the parameter values used in the simulations, and the justification for their selection, are reported in Table S1.

Supplementary Table S1: Model parameter values

Parameter	Value	Justification
$(s_0, i_0, d_0, a_0, e_0)$	$(1 - 10^{-5}, 10^{-5}, 0, 0, 0)$	Early stage of the pandemic
γ_i, γ_d	1/14 = 0.071	[10]
γ_a	1/12.4	[11]
β	0.251	$\overline{R}_0$ from [12]
ξ_i, ξ_d	0.0053	[13],[14]
μ	0.0085	[13]
$\bar{h}$	333×10^{-5}	[15]
$\hat{\mu}$	$5\times\mu$	[18]

Deceased population versus cost of government intervention

In Fig. 2 of the main text, we associate the cost of (optimal) government intervention and the portion of deceased population when: (i) different hospital capacity rates, (ii) different testing rates and (iii) different cost emphasis levels associated with the acutely symptomatic population, are considered. Note that in each case we considered a broad range of cost weight values associated to the total number of deaths. The considered cases for hospital capacity rates, testing rates, and cost weights attributed to the acutely symptomatic cases and the total number of deaths are presented in Table S2.

Supplementary Table S2: Parameters associated with the cases presented in Fig. 2 of the main text.

Parameter	Symbol	Cases
Hospital capacity rate	$\bar{h}$	$\{222, 333, 444\} \times 10^{-5}$
Testing rate	ν	$\{0, 0.05, 0.10\}$
Cost weight for acutely symptomatic cases	θ_a	$\{0, 5 \times 10^4, 10^5\}$
Cost weight for aggregate deaths	θ_e	$[0, 2.5 \times 10^4]$

The parameters from Table S2 associated with each of the 8 intervention strategies presented in Fig. 3 in the main text are provided in Table S3 below. In all cases, the considered hospital capacity rate was 333 care beds per 100,000 habitants which corresponded to the full capacity case described in the main text.

Supplementary Table S3: Parameters associated with the optimal continuous strategies presented in Fig. 3 of the main text.

Tolerance (%)	Testing rate	Cost weight for acutely symptomatic cases	Cost weight for aggregate deaths
1	0	0	1600
1	0.05	0	400
0.1	0	10^5	600
0.1	0.05	10^5	1000
0.1	0.10	5×10^4	1000
0.01	0	0	2.5×10^4
0.01	0.05	0	1.8×10^4
0.01	0.10	0	10^4

Implementing a limited number of policies and policy changes

In Supplementary Fig. S3–S10, we present the intervention strategies and corresponding aggregate deceases associated with decease tolerances of 1%, 0.1% and 0.01%. In particular, we present the optimal continuous strategy and optimized intervention strategies with $4, 7$ and 10 distinct policy levels and $6, 12$ and 18 policy changes respectively. These results are associated with Fig. 3 in the main text, which only includes the case where 4 distinct policies and 6 policy changes are allowed. The results presented in Supplementary Fig. S3–S10 demonstrate that increasing the number of distinct policies enables a decease response that is closer to the optimal continuous strategy, which is in agreement with intuition.

Effect of parametric uncertainty

In Supplementary Fig. S11–S26, we present additional results associated with the effect of uncertainty in knowledge of the initial reproduction number $\bar{R}_0$ and the infection fatality rate. These results aim to provide additional intuition and clarity on the findings presented in the main text.

In particular, we first consider the case where the value of $\bar{R}_0$ ranges between 3.17 and 3.38, which corresponds to a 95% confidence interval, following [12], when the infection mortality rate is 0.66%, as reported in [13]. For the considered case, we obtain the optimal strategies which correspond to each value of $\bar{R}_0$ for the parameters presented in Table S3, associated with the 8 continuous strategies presented in Fig. 3 in the main text. The ranges of the obtained optimal intervention strategies for each of these 8 considered cases are depicted in Supplementary Fig. S11–S18 (left). From these figures, it follows that the uncertainty in $\bar{R}_0$ results in small variations in the optimal strategies when no and slow testing policies are adopted. By contrast, when fast testing policies are implemented, the impact of the uncertainty in $\bar{R}_0$ on the optimal strategies is more substantial.

Moreover, Supplementary Fig. S11–S18 (right) demonstrate the ranges of decease rates obtained when the same 8 selected strategies are applied for $\bar{R}_0 \in [3.17, 3.38]$ when the infection mortality rate is 0.66%, i.e. they depict the decease rates from implementing the selected strategies when the value of $\bar{R}_0$ has been incorrectly estimated. The effect of implementing the 8 selected strategies when in addition the infection mortality rate has been incorrectly estimated is demonstrated in Supplementary Fig. S19–S26, which depict the decease rates for the considered range of values for $\bar{R}_0$ when the infection mortality rate is 0.39% and 1.33% respectively. These values represent the lower and upper bounds of a 95% infection mortality rate confidence interval, as reported in [13]. Supplementary Fig. S11–S18 (right) and Supplementary Fig. S19–S26 demonstrate that the level of the adopted decease tolerance is crucial when it comes to the effect of model uncertainty in the decease rates. The latter is in agreement with the results presented in the main text, which state that parametric uncertainty has a more substantial effect when stricter government strategies, associated with lower decease tolerances, are adopted.

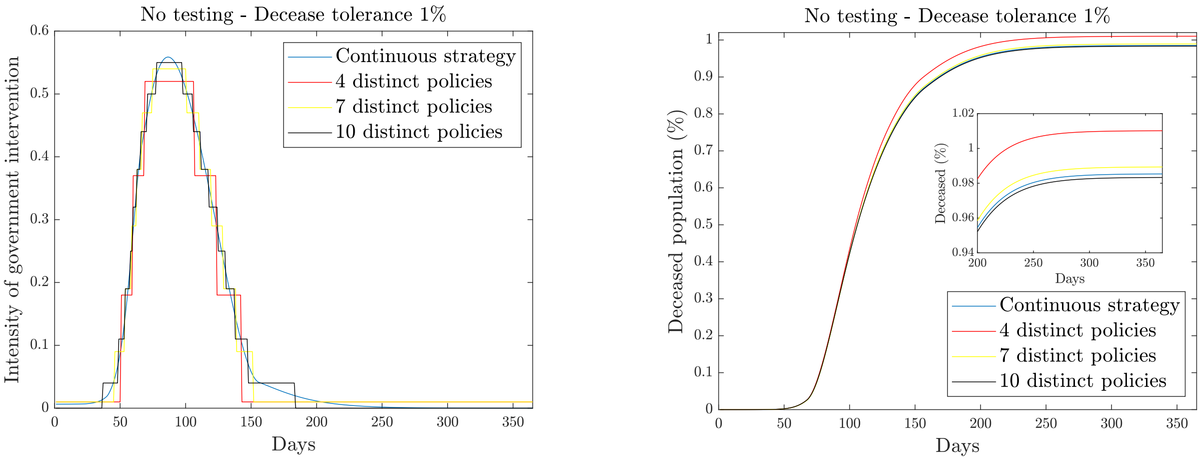

Supplementary Figure S3: **Intervention strategy and decease rate, 1% decease tolerance, no testing.** Intensity of government intervention (left) and portion of deceased population (right) when no testing is performed and a decease tolerance of 1% is adopted when (i) a continuously changing strategy is selected and (ii) discrete implementations of the selected strategy are considered, allowing $4, 7$ and 10 policy levels and $6, 12$ and 18 policy changes respectively.

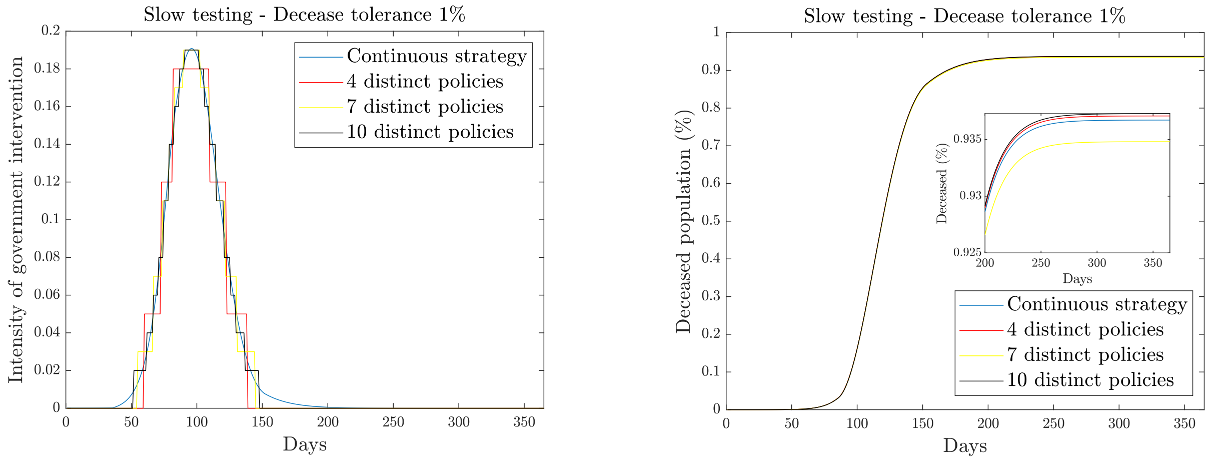

Supplementary Figure S4: **Intervention strategy and decease rate, 1% decease tolerance, slow testing.** Intensity of government intervention (left) and portion of deceased population (right) when a slow testing policy and a decease tolerance of 1% are adopted when (i) a continuously changing strategy is selected and (ii) discrete implementations of the selected strategy are considered, allowing $4, 7$ and 10 policy levels and $6, 12$ and 18 policy changes respectively.

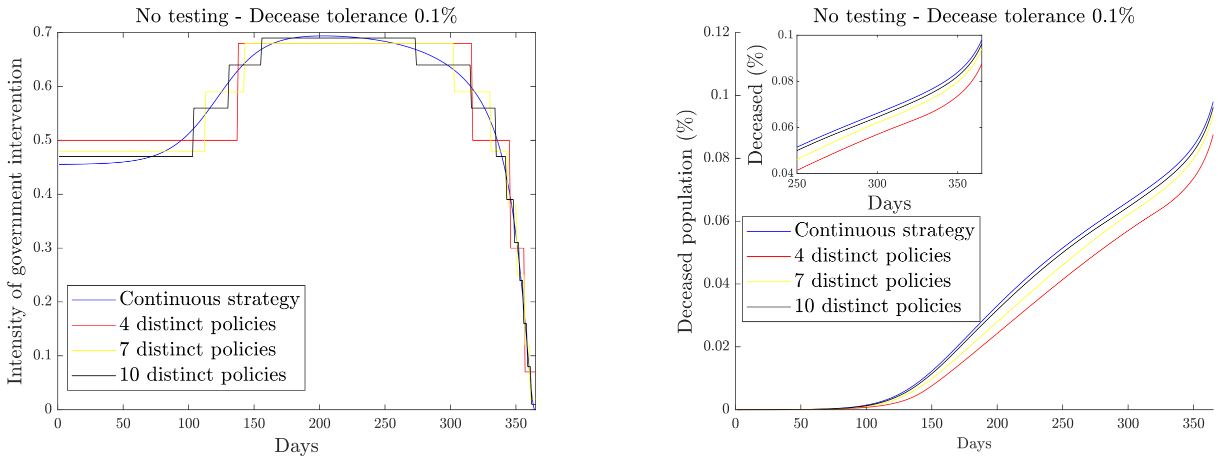

Supplementary Figure S5: **Intervention strategy and decease rate, 0.1% decease tolerance, no testing.** Intensity of government intervention (left) and portion of deceased population (right) when no testing is performed and a decease tolerance of 0.1% is adopted when (i) a continuously changing strategy is selected and (ii) discrete implementations of the selected strategy are considered, allowing $4, 7$ and 10 policy levels and $6, 12$ and 18 policy changes respectively.

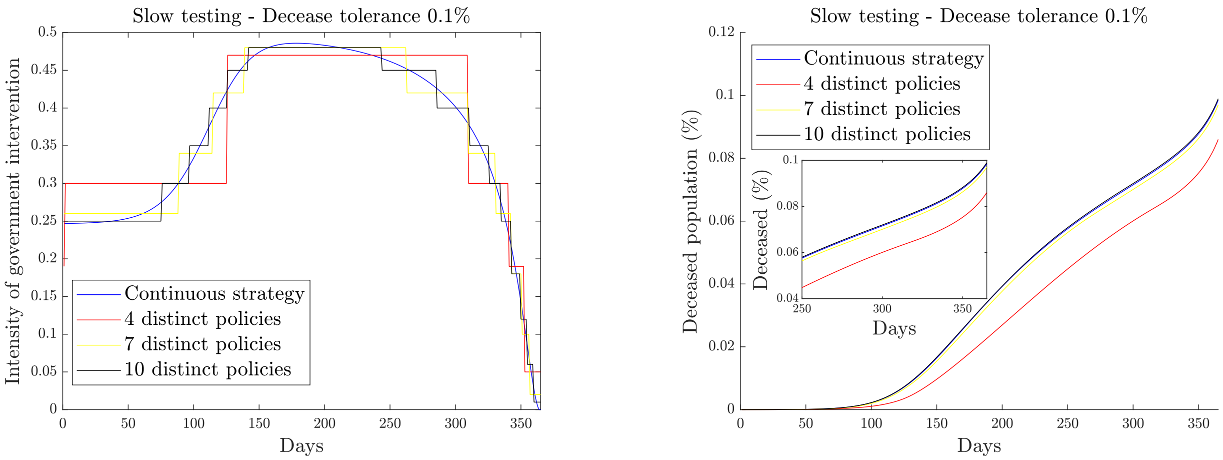

Supplementary Figure S6: **Intervention strategy and decease rate, 0.1% decease tolerance, slow testing.** Intensity of government intervention (left) and portion of deceased population (right) when a slow testing policy and a decease tolerance of 0.1% are adopted when (i) a continuously changing strategy is selected and (ii) discrete implementations of the selected strategy are considered, allowing $4, 7$ and 10 policy levels and $6, 12$ and 18 policy changes respectively.

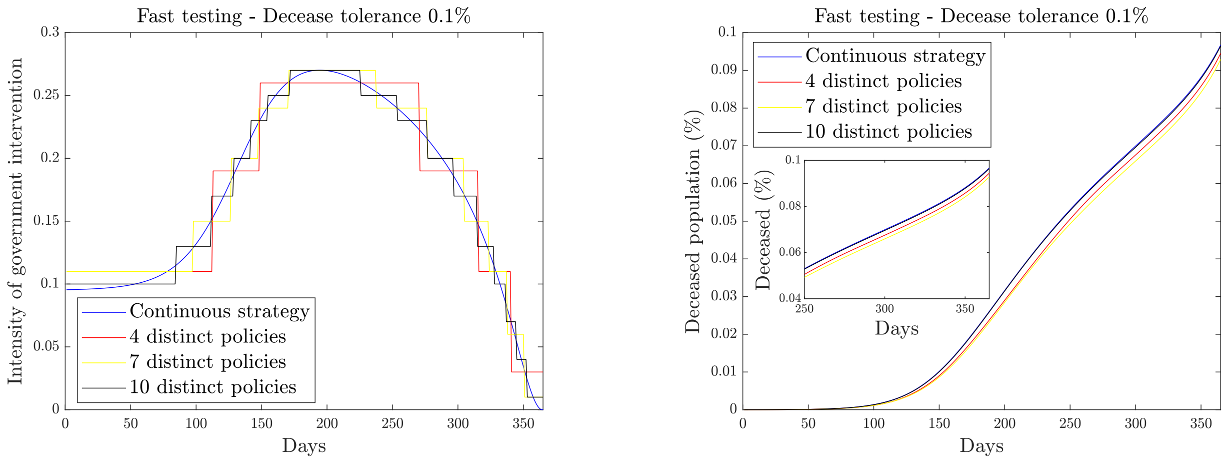

Supplementary Figure S7: **Intervention strategy and decease rate,** 0.1% **decease tolerance, fast testing.** Intensity of government intervention (left) and portion of deceased population (right) when a fast testing policy and a decease tolerance of 0.1% are adopted when (i) a continuously changing strategy is selected and (ii) discrete implementations of the selected strategy are considered, allowing $4, 7$ and 10 policy levels and $6, 12$ and 18 policy changes respectively.

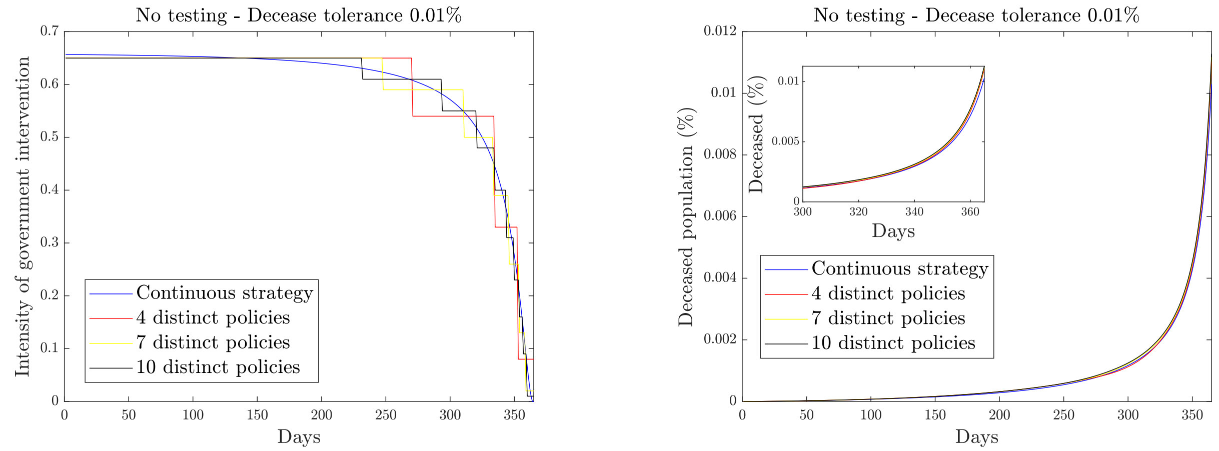

Supplementary Figure S8: **Intervention strategy and decease rate,** 0.01% **decease tolerance, no testing.** Intensity of government intervention (left) and portion of deceased population (right) when no testing is performed and a decease tolerance of 0.01% is adopted when (i) a continuously changing strategy is selected and (ii) discrete implementations of the selected strategy are considered, allowing $4, 7$ and 10 policy levels and $6, 12$ and 18 policy changes respectively.

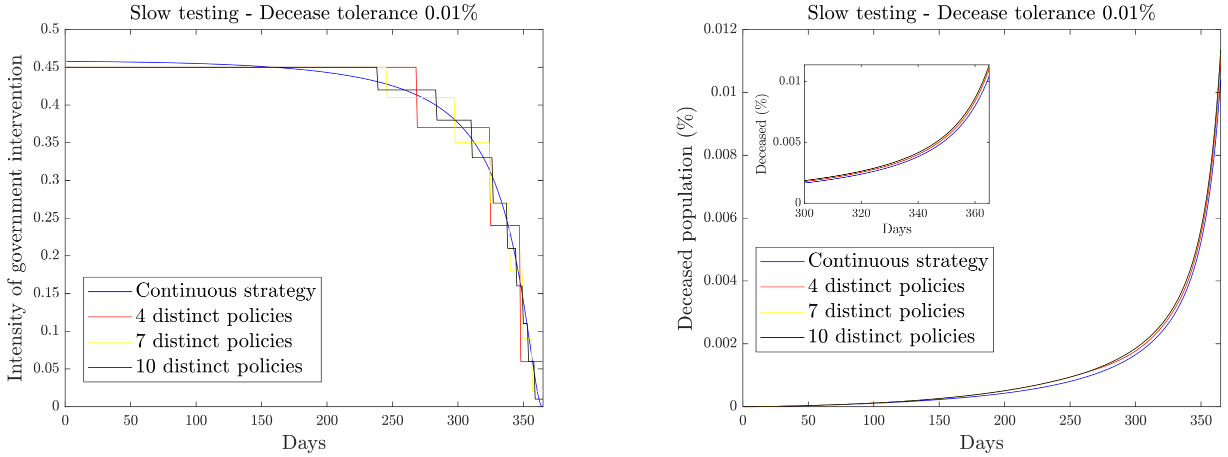

Supplementary Figure S9: **Intervention strategy and decease rate,** 0.01% **decease tolerance, slow testing.** Intensity of government intervention (left) and portion of deceased population (right) when a slow testing policy and a decease tolerance of 0.01% are adopted when (i) a continuously changing strategy is selected and (ii) discrete implementations of the selected strategy are considered, allowing $4, 7$ and 10 policy levels and $6, 12$ and 18 policy changes respectively.

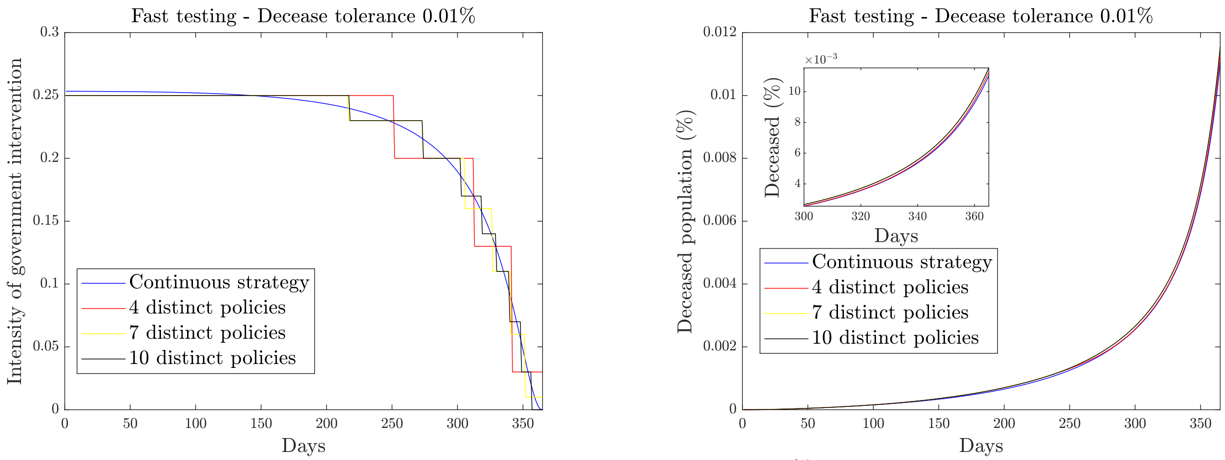

Supplementary Figure S10: **Intervention strategy and decease rate,** 0.01% **decease tolerance, fast testing.** Intensity of government intervention (left) and portion of deceased population (right) when a fast testing policy and a decease tolerance of 0.01% are adopted when (i) a continuously changing strategy is selected and (ii) discrete implementations of the selected strategy are considered, allowing $4, 7$ and 10 policy levels and $6, 12$ and 18 policy changes respectively.

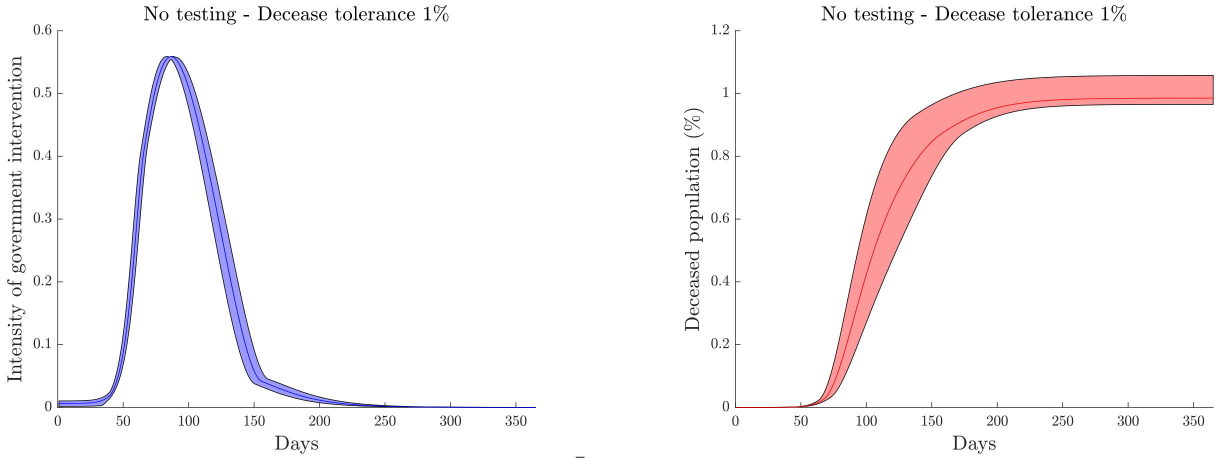

Supplementary Figure S11: **Effect of uncertainty in $\bar{R}_0$ on optimal strategy and decease rate,** 1% **decease tolerance, no testing.** Both subfigures consider the range $\bar{R}_0 \in [3.17, 3.38]$, a no testing policy and a decease tolerance of 1%. (left) Ranges of optimal strategies, (right) Ranges of aggregate deceases when the optimal strategy obtained based on $\bar{R}_0 = 3.27$ and infection mortality rate of 0.66% is implemented. The darker line corresponds to $\bar{R}_0 = 3.27$.

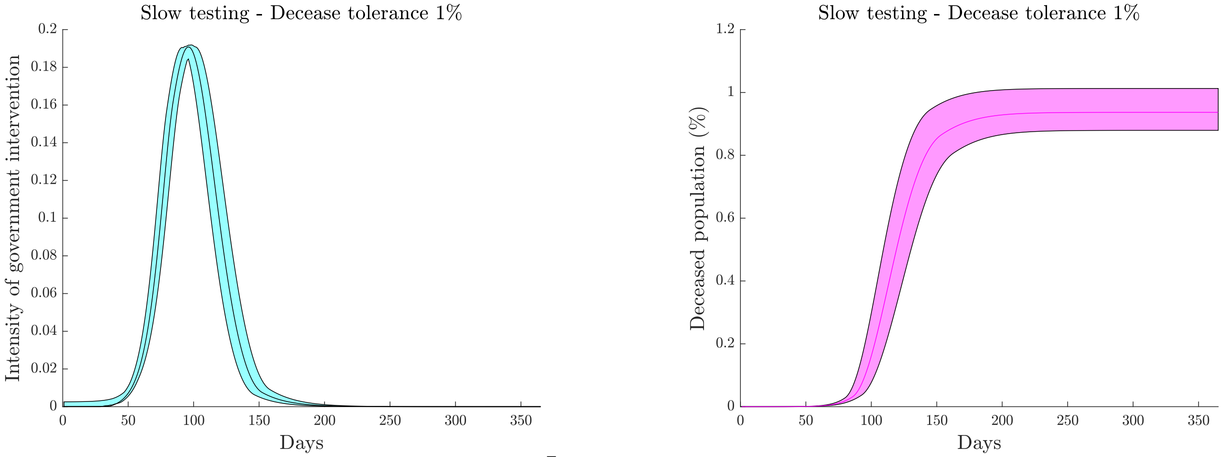

Supplementary Figure S12: **Effect of uncertainty in $\bar{R}_0$ on optimal strategy and decease rate,** 1% **decease tolerance, slow testing.** Both subfigures consider the range $\bar{R}_0 \in [3.17, 3.38]$, a slow testing policy and a decease tolerance of 1%. (left) Ranges of optimal strategies, (right) Ranges of aggregate deceases when the optimal strategy obtained based on $\bar{R}_0 = 3.27$ and infection mortality rate of 0.66% is implemented. The darker line corresponds to $\bar{R}_0 = 3.27$.

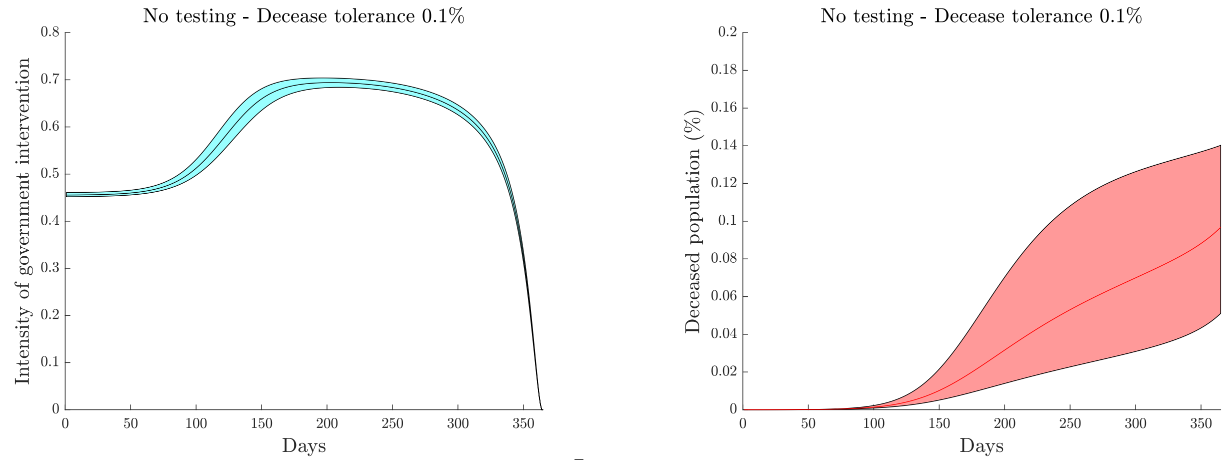

Supplementary Figure S13: **Effect of uncertainty in $\bar{R}_0$ on optimal strategy and decease rate,** 0.1% **decease tolerance, no testing.** Both subfigures consider the range $\bar{R}_0 \in [3.17, 3.38]$, a no testing policy and a decease tolerance of 0.1%. (left) Ranges of optimal strategies, (right) Ranges of aggregate deceases when the optimal strategy obtained based on $\bar{R}_0 = 3.27$ and infection mortality rate of 0.66% is implemented. The darker line corresponds to $\bar{R}_0 = 3.27$.

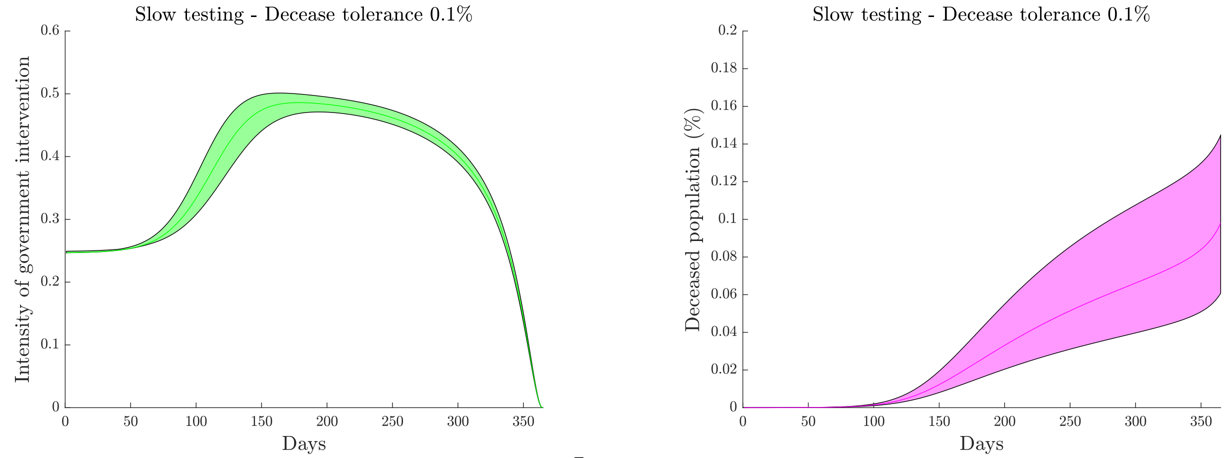

Supplementary Figure S14: **Effect of uncertainty in $\bar{R}_0$ on optimal strategy and decease rate,** 0.1% **decease tolerance, slow testing.** Both subfigures consider the range $\bar{R}_0 \in [3.17, 3.38]$, a slow testing policy and a decease tolerance of 0.1%. (left) Ranges of optimal strategies, (right) Ranges of aggregate deceases when the optimal strategy obtained based on $\bar{R}_0 = 3.27$ and infection mortality rate of 0.66% is implemented. The darker line corresponds to $\bar{R}_0 = 3.27$.

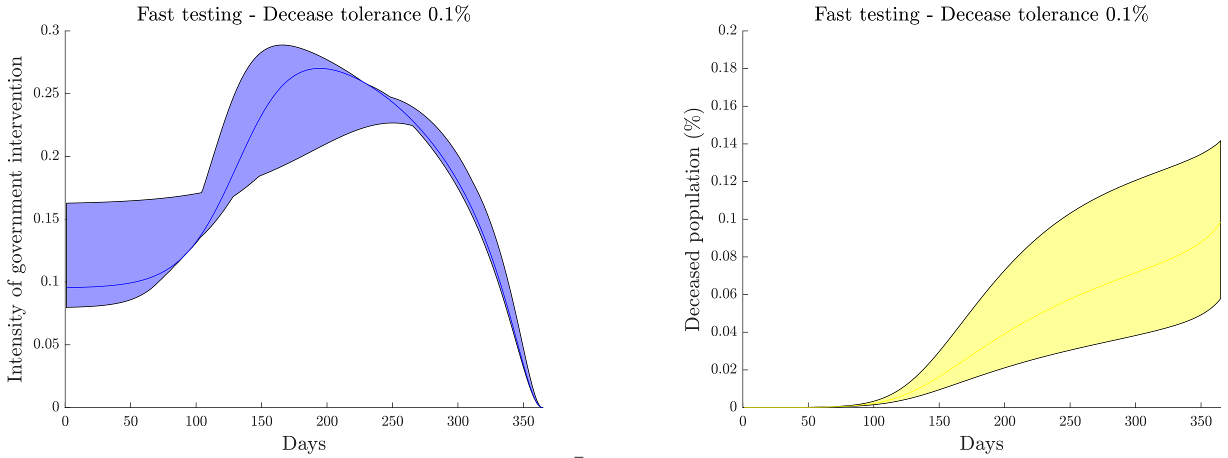

Supplementary Figure S15: **Effect of uncertainty in $\bar{R}_0$ on optimal strategy and decease rate,** 0.1% **decease tolerance, fast testing.** Both subfigures consider the range $\bar{R}_0 \in [3.17, 3.38]$, a fast testing policy and a decease tolerance of 0.1%. (left) Ranges of optimal strategies, (right) Ranges of aggregate deceases when the optimal strategy obtained based on $\bar{R}_0 = 3.27$ and infection mortality rate of 0.66% is implemented. The darker line corresponds to $\bar{R}_0 = 3.27$.

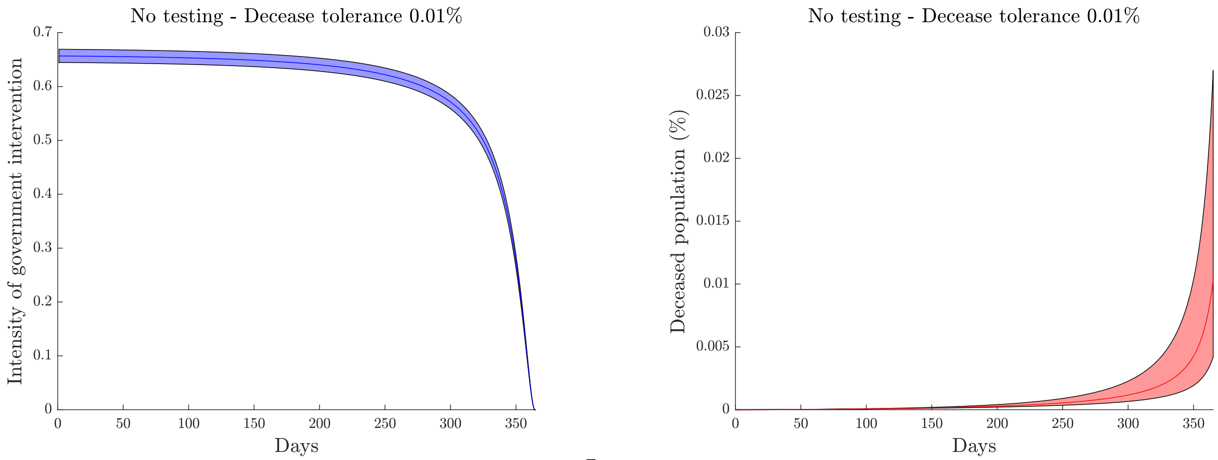

Supplementary Figure S16: **Effect of uncertainty in $\bar{R}_0$ on optimal strategy and decease rate,** 0.01% **decease tolerance, no testing.** Both subfigures consider the range $\bar{R}_0 \in [3.17, 3.38]$, a no testing policy and a decease tolerance of 0.01%. (left) Ranges of optimal strategies, (right) Ranges of aggregate deceases when the optimal strategy obtained based on $\bar{R}_0 = 3.27$ and infection mortality rate of 0.66% is implemented. The darker line corresponds to $\bar{R}_0 = 3.27$.

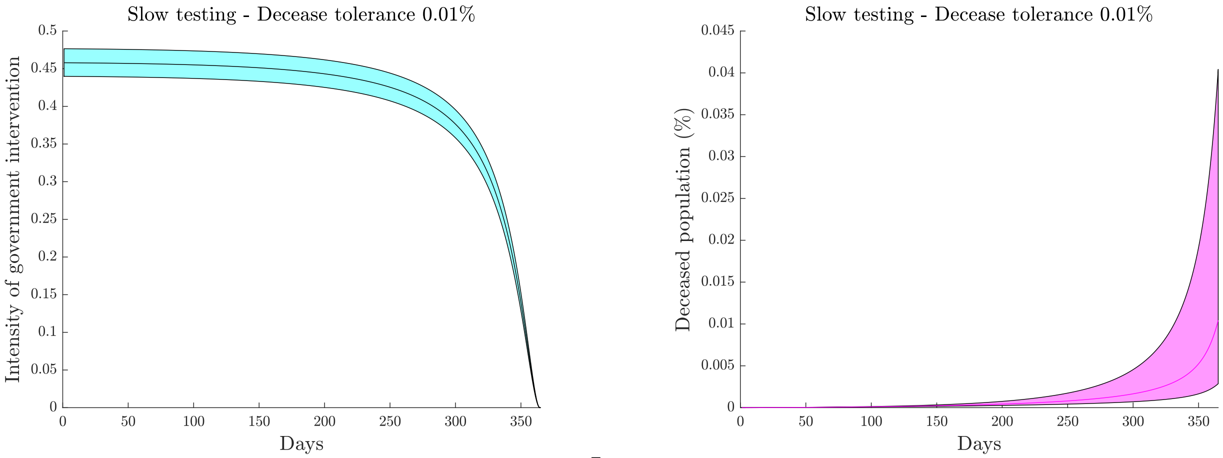

Supplementary Figure S17: **Effect of uncertainty in $\bar{R}_0$ on optimal strategy and decease rate,** 0.01% **decease tolerance, slow testing.** Both subfigures consider the range $\bar{R}_0 \in [3.17, 3.38]$, a slow testing policy and a decease tolerance of 0.01%. (left) Ranges of optimal strategies, (right) Ranges of aggregate deceases when the optimal strategy obtained based on $\bar{R}_0 = 3.27$ and infection mortality rate of 0.66% is implemented. The darker line corresponds to $\bar{R}_0 = 3.27$.

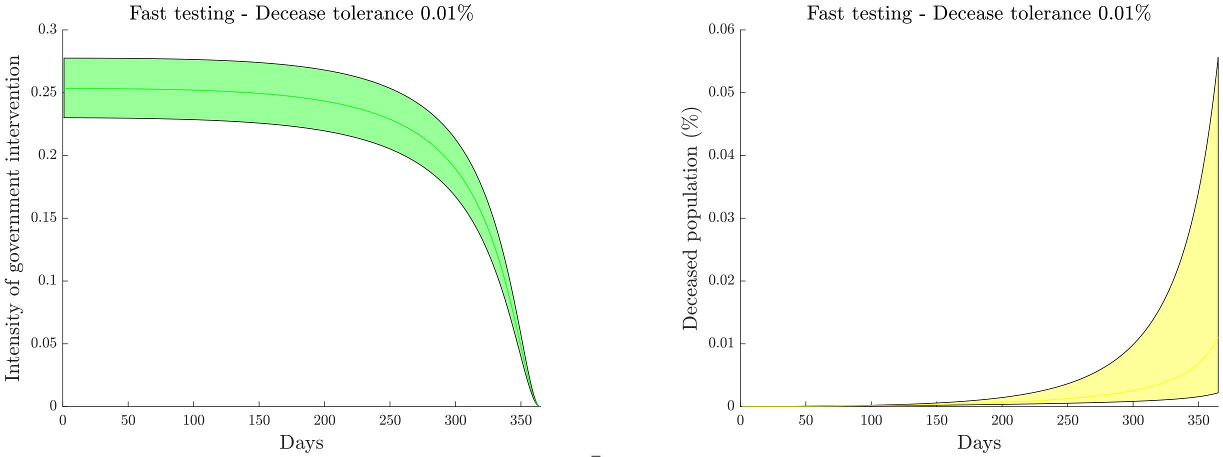

Supplementary Figure S18: **Effect of uncertainty in $\bar{R}_0$ on optimal strategy and decease rate,** 0.01% **decease tolerance, fast testing.** Both subfigures consider the range $\bar{R}_0 \in [3.17, 3.38]$, a fast testing policy and a decease tolerance of 0.01%. (left) Ranges of optimal strategies, (right) Ranges of aggregate deceases when the optimal strategy obtained based on $\bar{R}_0 = 3.27$ and infection mortality rate of 0.66% is implemented. The darker line corresponds to $\bar{R}_0 = 3.27$.

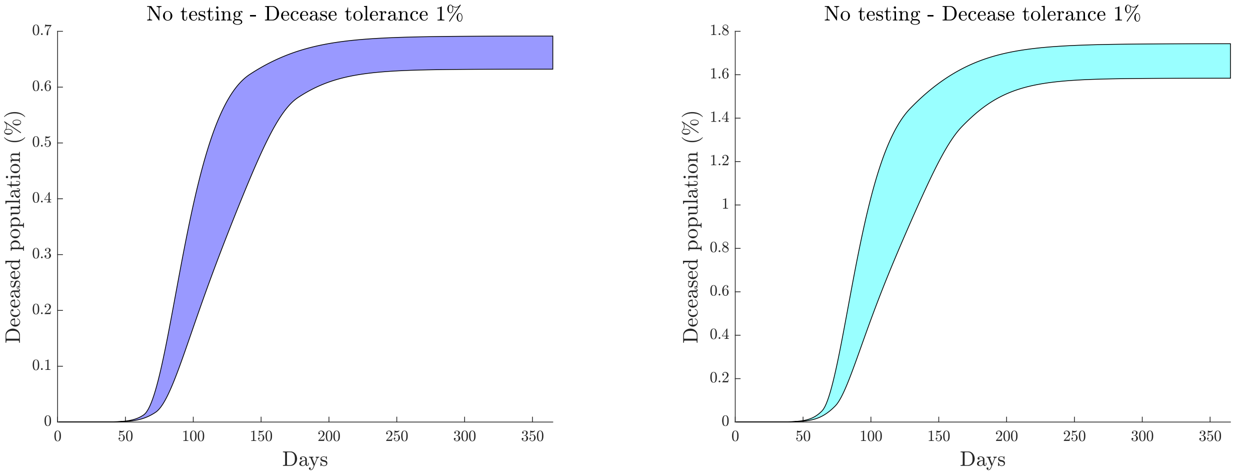

Supplementary Figure S19: **Combined effect of uncertainty in $\bar{R}_0$ and infection fatality rate on the decease rate, 1% decease tolerance, no testing.** Ranges of aggregate deceases for $\bar{R}_0 \in [3.17, 3.38]$ when no testing is performed and a decease tolerance of 1% is adopted with infection mortality rates of 0.39% (left) and 1.33% (right) when the optimal strategy obtained based on $\bar{R}_0 = 3.27$ and infection mortality rate of 0.66% is implemented.

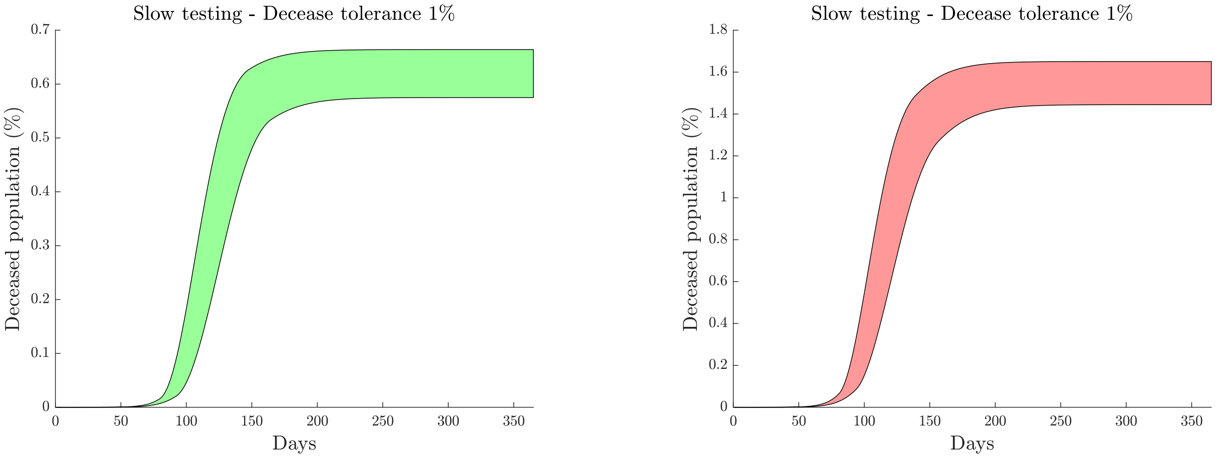

Supplementary Figure S20: **Combined effect of uncertainty in $\bar{R}_0$ and infection fatality rate on the decease rate, 1% decease tolerance, slow testing.** Ranges of aggregate deceases for $\bar{R}_0 \in [3.17, 3.38]$ when a slow testing policy and a decease tolerance of 1% are adopted with infection mortality rates of 0.39% (left) and 1.33% (right) when the optimal strategy obtained based on $\bar{R}_0 = 3.27$ and infection mortality rate of 0.66% is implemented.

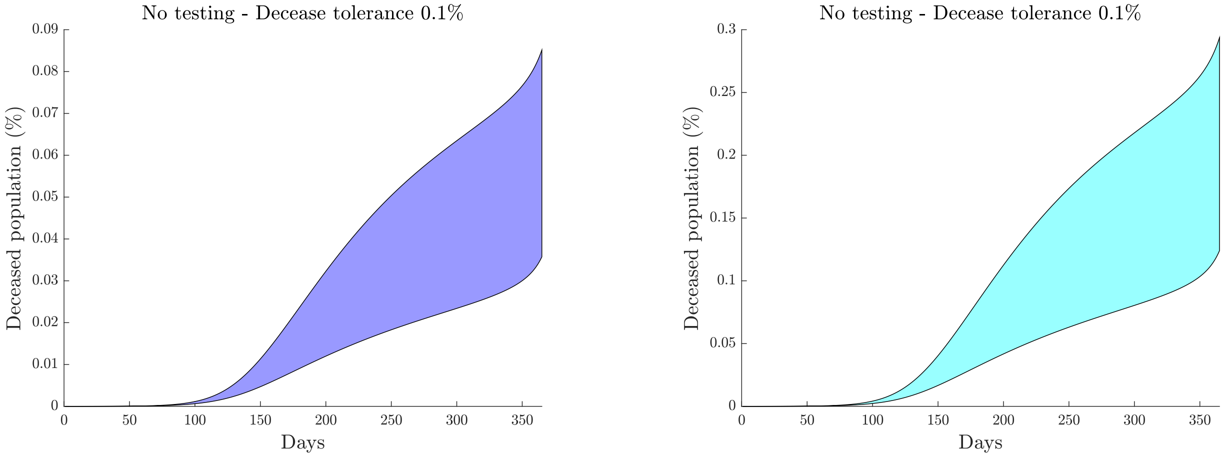

Supplementary Figure S21: **Combined effect of uncertainty in $\bar{R}_0$ and infection fatality rate on the decease rate, 0.1% decease tolerance, no testing.** Ranges of aggregate deceases for $\bar{R}_0 \in [3.17, 3.38]$ when no testing is performed and a decease tolerance of 0.1% is adopted with infection mortality rates of 0.39% (left) and 1.33% (right) when the optimal strategy obtained based on $\bar{R}_0 = 3.27$ and infection mortality rate of 0.66% is implemented.

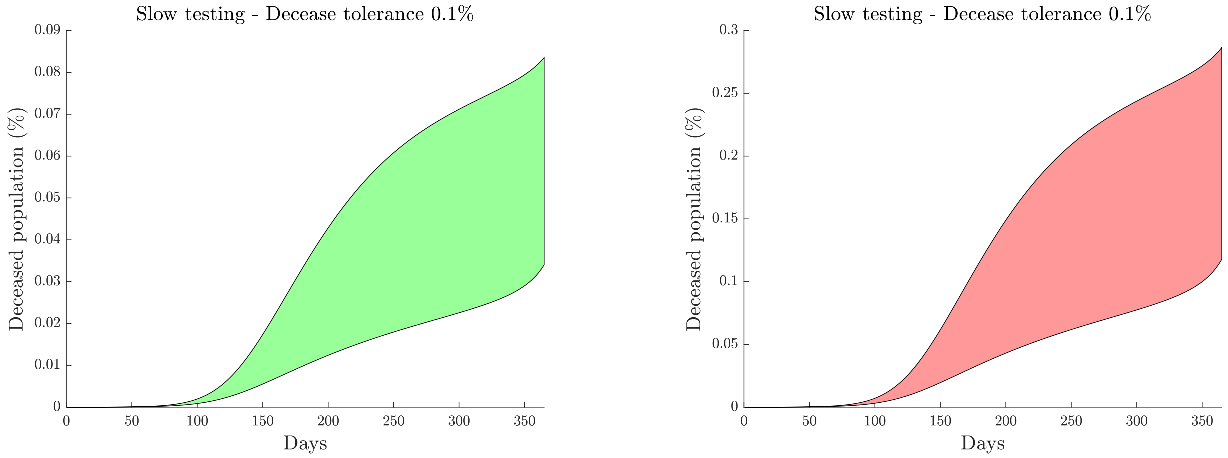

Supplementary Figure S22: **Combined effect of uncertainty in $\bar{R}_0$ and infection fatality rate on the decease rate, 0.1% decease tolerance, slow testing.** Ranges of aggregate deceases for $\bar{R}_0 \in [3.17, 3.38]$ when a slow testing policy and a decease tolerance of 0.1% are adopted with infection mortality rates of 0.39% (left) and 1.33% (right) when the optimal strategy obtained based on $\bar{R}_0 = 3.27$ and infection mortality rate of 0.66% is implemented.

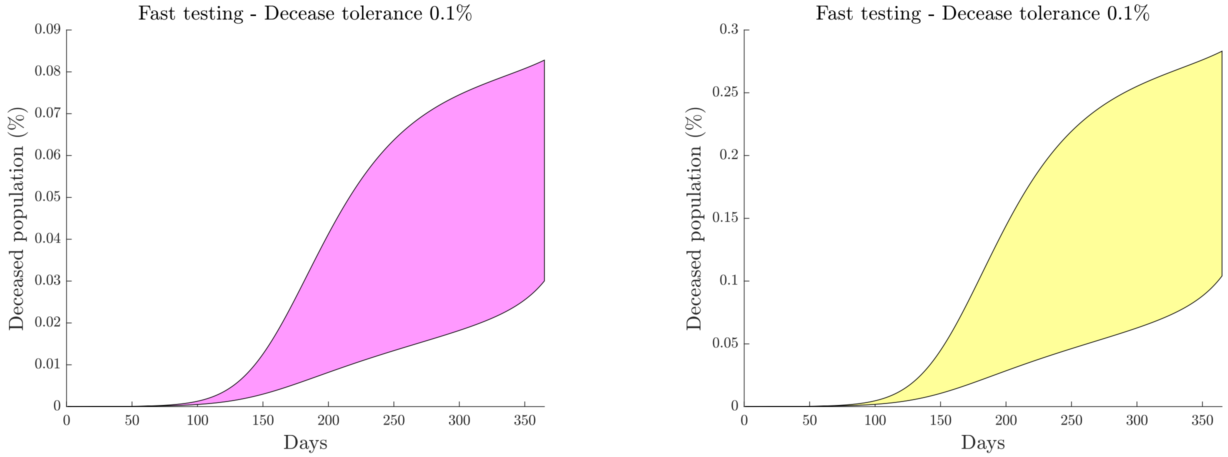

Supplementary Figure S23: **Combined effect of uncertainty in $\bar{R}_0$ and infection fatality rate on the decease rate, 0.1% decease tolerance, fast testing.** Ranges of aggregate deceases for $\bar{R}_0 \in [3.17, 3.38]$ when a fast testing policy and a decease tolerance of 0.1% are adopted with infection mortality rates of 0.39% (left) and 1.33% (right) when the optimal strategy obtained based on $\bar{R}_0 = 3.27$ and infection mortality rate of 0.66% is implemented.

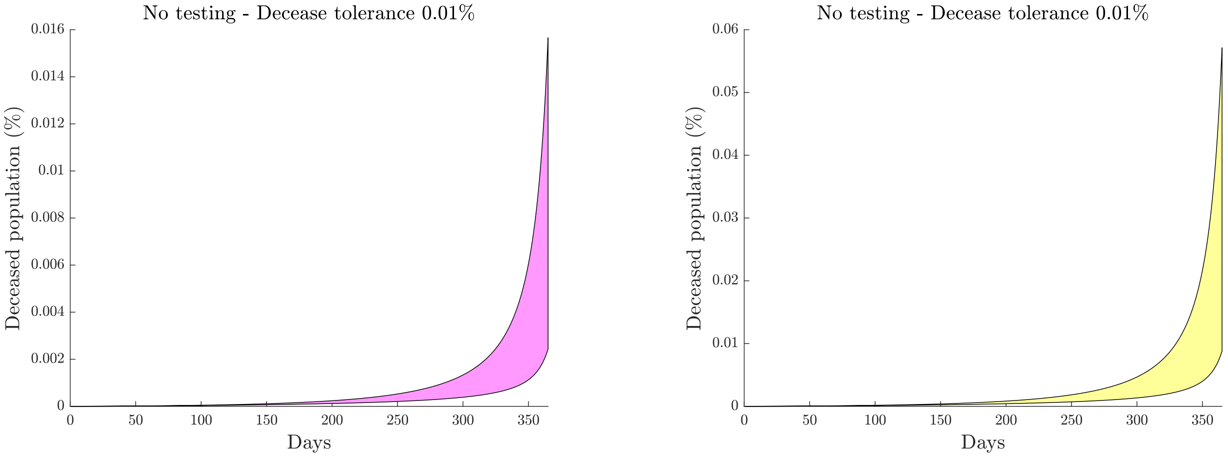

Supplementary Figure S24: **Combined effect of uncertainty in $\bar{R}_0$ and infection fatality rate on the decease rate, 0.01% decease tolerance, no testing.** Ranges of aggregate deceases for $\bar{R}_0 \in [3.17, 3.38]$ when no testing is performed and a decease tolerance of 0.01% is adopted with infection mortality rates of 0.39% (left) and 1.33% (right) when the optimal strategy obtained based on $\bar{R}_0 = 3.27$ and infection mortality rate of 0.66% is implemented.

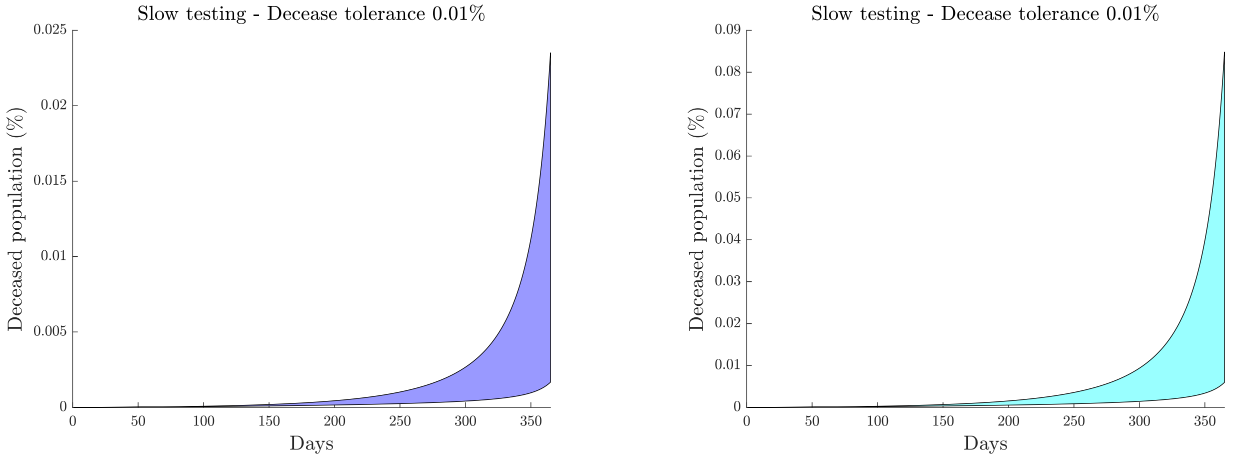

Supplementary Figure S25: **Combined effect of uncertainty in $\bar{R}_0$ and infection fatality rate on the decease rate, 0.01% decease tolerance, slow testing.** Ranges of aggregate deceases for $\bar{R}_0 \in [3.17, 3.38]$ when a slow testing policy and a decease tolerance of 0.01% are adopted with infection mortality rates of 0.39% (left) and 1.33% (right) when the optimal strategy obtained based on $\bar{R}_0 = 3.27$ and infection mortality rate of 0.66% is implemented.

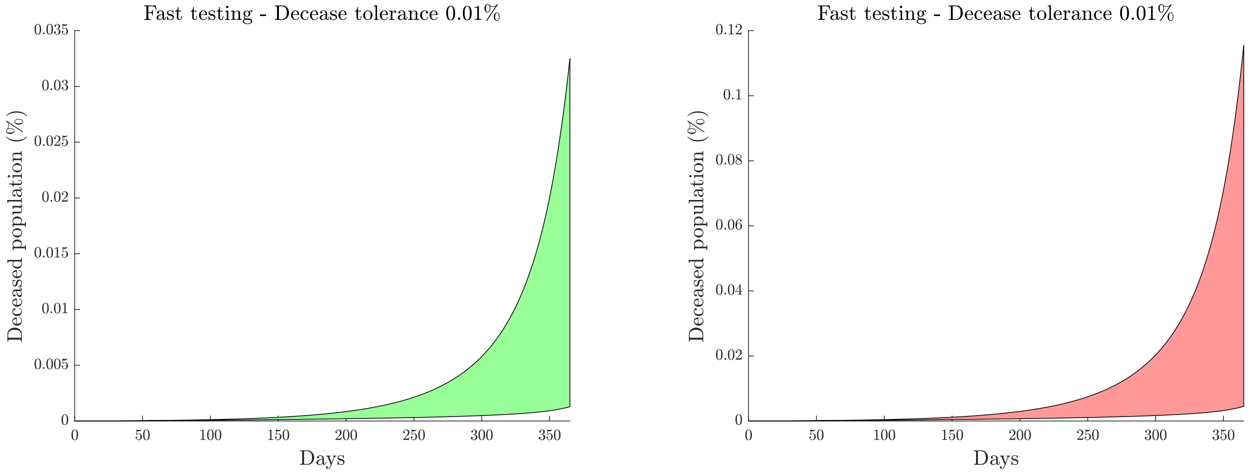

Supplementary Figure S26: **Combined effect of uncertainty in $\bar{R}_0$ and infection fatality rate on the decease rate, 0.01% decease tolerance, fast testing.** Ranges of aggregate deceases for $\bar{R}_0 \in [3.17, 3.38]$ when a fast testing policy and a decease tolerance of 0.01% are adopted with infection mortality rates of 0.39% (left) and 1.33% (right) when the optimal strategy obtained based on $\bar{R}_0 = 3.27$ and infection mortality rate of 0.66% is implemented.

Supplementary References

[1] Khalil, H. K. *Nonlinear systems*, vol. 3 (Prentice Hall New Jersey, 1996).

[2] Haddad, W. M. & Chellaboina, V. *Nonlinear dynamical systems and control: a Lyapunov-based approach* (Princeton university press, 2011).

[3] Flaxman, S. *et al.* Estimating the effects of non-pharmaceutical interventions on COVID-19 in Europe. *Nature* (2020).

[4] Fleming, W. H. & Rishel, R. W. *Deterministic and stochastic optimal control*, vol. 1 (Springer Science & Business Media, 2012).

[5] Pontryagin, L. S. *Mathematical theory of optimal processes* (Routledge, 2018).

[6] Rockafellar, R. T. *Convex analysis* (Princeton university press, 2015).

[7] Filippov, A. *Differential Equations with Discontinuous Righthand Sides: Control Systems*, vol. 18 (Springer Science & Business Media, 1988).

[8] Lenhart, S. & Workman, J. T. *Optimal control applied to biological models* (CRC press, 2007).

[9] Abbott, S. *Understanding analysis*, vol. 2 (Springer, 2001).

[10] World Health Organization. Report of the WHO - China joint mission on coronavirus disease 2019 (COVID-19) (2020).

[11] Wang, H. *et al.* Phase-adjusted estimation of the number of coronavirus disease 2019 cases in Wuhan, China. *Cell discovery* **6**, 1–8 (2020).

[12] Yuan, J., Li, M., Lv, G. & Lu, Z. K. Monitoring transmissibility and mortality of COVID-19 in Europe. *International Journal of Infectious Diseases* (2020).

[13] Verity, R., Okell, L. C., Dorigatti, I. *et al.* Estimates of the severity of coronavirus disease 2019: a model-based analysis. *The Lancet infectious diseases* (2020).

[14] United Nations, Department of Economic and Social Affairs. Population data (Retrieved on 30/11/2020).

[15] Rhodes, A. *et al.* The variability of critical care bed numbers in Europe. *Intensive care medicine* **38**, 1647–1653 (2012).

[16] Mallapaty, S. How deadly is the coronavirus? Scientists are close to an answer. *Nature* **582**, 467–468 (2020).

[17] Salje, H. *et al.* Estimating the burden of SARS-CoV-2 in France. *Science* (2020).

[18] Catena, R. & Holweg, M. We need to relocate ICU patients out of COVID-19 hotspots. *Harvard Business Review* (2020).
